# Recombination-aware phylogeographic inference using the structured coalescent with ancestral recombination

**DOI:** 10.1371/journal.pcbi.1010422

**Published:** 2022-08-19

**Authors:** Fangfang Guo, Ignazio Carbone, David A. Rasmussen

**Affiliations:** 1 Department of Entomology and Plant Pathology, North Carolina State University, Raleigh, North Carolina, United States of America; 2 Center for Integrated Fungal Research, North Carolina State University, Raleigh, North Carolina, United States of America; 3 Bioinformatics Research Center, North Carolina State University, Raleigh, North Carolina, United States of America; Max Planck Institute For Evolutionary Anthropology, GERMANY

## Abstract

Movement of individuals between populations or demes is often restricted, especially between geographically isolated populations. The structured coalescent provides an elegant theoretical framework for describing how movement between populations shapes the genealogical history of sampled individuals and thereby structures genetic variation within and between populations. However, in the presence of recombination an individual may inherit different regions of their genome from different parents, resulting in a mosaic of genealogical histories across the genome, which can be represented by an Ancestral Recombination Graph (ARG). In this case, different genomic regions may have different ancestral histories and so different histories of movement between populations. Recombination therefore poses an additional challenge to phylogeographic methods that aim to reconstruct the movement of individuals from genealogies, although also a potential benefit in that different loci may contain additional information about movement. Here, we introduce the Structured Coalescent with Ancestral Recombination (SCAR) model, which builds on recent approximations to the structured coalescent by incorporating recombination into the ancestry of sampled individuals. The SCAR model allows us to infer how the migration history of sampled individuals varies across the genome from ARGs, and improves estimation of key population genetic parameters such as population sizes, recombination rates and migration rates. Using the SCAR model, we explore the potential and limitations of phylogeographic inference using full ARGs. We then apply the SCAR to lineages of the recombining fungus *Aspergillus flavus* sampled across the United States to explore patterns of recombination and migration across the genome.

## Author summary

Phylogeographic methods are widely used to reconstruct the historical movement of individuals between different populations. When applied to infectious pathogens, these methods are often used to reconstruct the origin or source of novel pathogen lineages. Most existing phylogeographic methods reconstruct movement based on a single phylogenetic tree, which is assumed to reflect the genetic ancestry of all sampled individuals. However in populations undergoing recombination, genetic material can be exchanged between lineages such that individuals may inherit different regions of their genome from different ancestors. In this case, phylogenetic relationships among individuals can only be captured by a reticulated network rather than any single tree. Ancestral Recombination Graphs (ARGs) provide one way of capturing these reticulate relationships and we develop new models that allow for demographic inference of historical population sizes, recombination rates and migration rates between subpopulations from ARGs. By accounting for recombination, our models not only allow for accurate demographic inference, but can take full advantage of the additional information contained in ARGs about how ancestry varies across genomes to more precisely reconstruct the movement of genetic material between populations.

## Introduction

In the absence of any recombination, populations evolve clonally and the ancestral relationships among all individuals can be captured by a single genealogy or phylogenetic tree [1, 2]. However, in the presence of recombination, individuals can inherit different parts of their genome from different ancestors, leading to a mosaic of phylogenetic relationships across the genome that cannot be captured by any single tree. Since many population genetic and phylogeographic methods infer demographic parameters (e.g. population sizes, migration rates) from a phylogeny assumed to reflect the clonal ancestry of sampled individuals, recombination poses a major challenge to demographic inference.

Rates of recombination vary dramatically from asexual populations that experience no recombination to sexually outcrossing populations where recombination occurs between parental genomes every generation [3–5]. How demographic inference methods deal with recombination largely depends on the assumed rate of recombination. If recombination rates are very low, individuals will inherit large regions of their genome (i.e. non-recombinant blocks) from the same set of ancestors. Moreover, recombination will only impact the ancestry of lineages directly involved in a recombination event while preserving the ancestral relationships among non-recombinant lineages [2]. In this case, phylogenies can be reconstructed from non-recombinant regions of the genome or recombining lineages can be identified and removed. At the other extreme, very high rates of recombination will break apart linkage between loci, such that different loci can be treated independently [6]. In this case population genomic methods that treat each locus as (pseudo-)independent can be used to draw demographic inferences [7].

However, in between these two extremes lie many organisms that undergo intermediate amounts of recombination, including many important microbial pathogens [8]. For example, this includes many fungi with mixed mating systems that are predominately clonal but occasionally reproduce sexually and thus recombine [9]. Such intermediate rates of recombination pose a particular challenge to demographic inference as it may be difficult to identify and accurately reconstruct phylogenetic relationships from any non-recombinant genomic region. At the same time, recombination is not frequent enough to breakdown correlations among linked loci, violating assumptions of independence between loci and simply concatenating alignments may lead to phylogenetic reconstructions inconsistent with the true ancestry of the sample [10].

Ideally, the differing but correlated patterns of ancestry across the genome would be captured using ancestral recombination graphs (ARGs) [11]. An ARG describes the complete genealogical history of sampled individuals, including *local* trees representing the genealogy of sampled individuals over a particular non-recombinant region of the genome and the recombination events connecting lineages across local trees. Although reconstructing full ARGs is notoriously difficult, recent advances now allow ARGs to be accurately reconstructed for a modest number of samples (i.e. <100). Notably, ARGweaver [12] allows for full Bayesian inference of ARGs under the sequential Markov coalescent (SMC) model, an approximation to the full coalescent with recombination [13]. More recent methods allow for ARGs to be approximated for much larger datasets as a series of correlated local or marginal trees [7, 14]. These methods however generally do not reconstruct the recombination events required to explain the topological differences between local trees.

In addition to recombination, population structure can also strongly shape the genealogical history of a population. The structured coalescent extends basic coalescent models by allowing lineages to migrate between different subpopulations or demes [15]. While the structured coalescent is most often used to model geographic structure, the theory holds for many different forms of population structure (e.g. assortative mating within a population) [16]. Under the structured coalescent, migration rates can be estimated from a genealogy of individuals sampled from different populations [17], and structured coalescent models form the basis of several phylogeographic inference frameworks [18–20]. However, population structure also poses a major challenge to demographic inference under coalescent models because lineages in the genealogy are no longer exchangeable in the sense that the probability of two lineages coalescing will depend on their ancestral location or state. Statistical inference under the structured coalescent therefore requires the ancestral state of lineages to be imputed, and early methods implemented algorithms to sample ancestral states using Markov chain Monte Carlo (MCMC) or other sampling-based methods [17]. Because jointly estimating the ancestral locations of all lineages along with the demographic parameters of interest poses yet another computational challenge, more recent methods make various approximations to the full structured coalescent to track the movement of lineages probabilistically, such that the unknown ancestral locations can be marginalized or integrated over [21–23].

Given that the statistical and computational performance of ARG reconstruction methods continue to improve at a rapid pace [24, 25], we explore phylogeographic inference where the ARG is assumed to be known or at least reconstructed accurately. We first develop a new model we call the Structured Coalescent with Ancestral Recombination (SCAR) to estimate demographic parameters in the presence of both migration and recombination from a reconstructed ARG. In essence, the SCAR model extends Hudson’s Coalescent with Recombination model [6] to include migration by using approximations to the structured coalescent that marginalize over unknown ancestral states [21–23]. Next, we test the limits of reconstructing ARGs from genomic sequence data using ARGweaver and then explore how accurately demographic parameters can be inferred from reconstructed ARGs using the SCAR model. Using simulated sequence data, we show that parameters such as recombination rates, migration rates and population sizes can be accurately estimated from ARGs under the SCAR model as long as the underlying ARG can be accurately reconstructed. We then apply this approach to the plant fungal pathogen *Aspergillus flavus* to estimate recombination and migration rates between natural populations in several US states.

## Models and methods

### The SCAR model

#### Description of the SCAR model

Here we incorporate recombination and population structure simultaneously into the coalescent process. We consider a population divided into *q* different demes or (sub)populations. Each population *k* is composed of *N*_*k*_ haploid individuals which reproduce each generation to generate a random number of offspring (i.e. a Wright-Fisher population). Two lineages can exchange genetic material through recombination, in which case their children may inherit genetic material from both parents. Lastly, we allow individuals to transition or migrate between populations.

**Fig 1:**
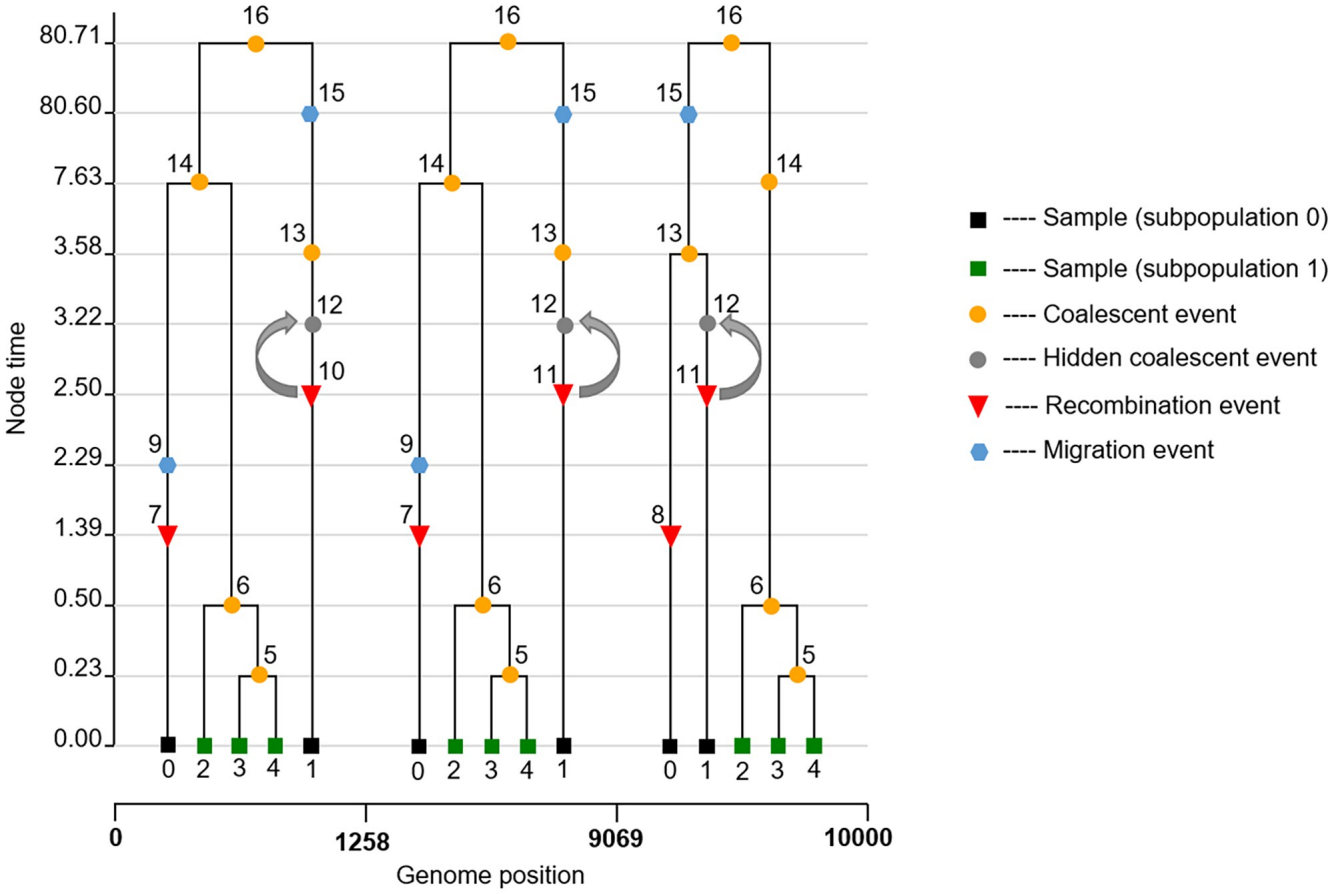
An ancestral recombination graph. In this example, an ARG was simulated for five individuals sampled in two subpopulations (0 and 1) using msprime [26]. Two recombination events happen, dividing the genome into three segments each with their own local tree. The first and second local tree have the same topology, because after the recombination event (indicated by nodes 10 and 11) the two parent lineages coalesce with one another (indicated by the grey arrow) at a hidden coalescent event (indicated by node 12), which would not normally be observed in the local trees. The second and the third local tree are topologically discordant due to a recombination event (indicated by nodes 7 and 8).

Three different types of events can therefore occur in the ancestry of sampled lineages under this model: coalescent events, recombination events and migration events (see Fig 1). We begin by considering the rate at which each one of these events will occur among lineages in the genealogy.

**Coalescent events**: As under the standard coalescent for a Wright-Fisher population [27, 28], the probability of two lineages finding their most recent common ancestor in a given generation is inversely proportional to the population size. Pairs of lineages in population *k* will therefore coalesce at rate $\lambda_{k}=\frac{1}{N_{k}}$ per generation. For now, we will assume lineages in different populations cannot coalesce, although this assumption can be relaxed (see for example Volz [21]). The total coalescent rate among all pairs of lineages in population *k* is (ak2)λk, where *a*_*k*_ is the number of lineages in *k*. For now we will also assume the ancestral location of lineages is known.

**Recombination events**: As in earlier models for the coalescent with recombination, each lineage in the tree undergoes a recombination event at rate *r* per site along a genome of length *L* [6, 26, 29]. However, because two parents contribute genetic material to a child lineage at a recombination event, not all of the genetic material each parent lineage carries will be ancestral to the sample [6]. We therefore need to track which sites in a lineage’s genome are ancestral or non-ancestral to the sample to determine whether or not a recombination event will impact the genealogy of the sample.

Following Kuhner et al. [29], we use the term *eligible links* to refer to sites that are eligible to undergo recombination because they separate two or more sites destined to contribute genetic material to the sample. While recombination events in regions of non-ancestral material will generally have no effect on the genealogy of sampled individuals, ancestral material may be separated by regions of *trapped* non-ancestral material [30] (S1 Fig), and recombination events occurring within trapped non-ancestral material may also impact the genealogy of the sample by splitting ancestral material to the left and right of the breakpoint onto different parental genomes. If we define *l*_*min*_ as the leftmost position and *r*_*max*_ as the rightmost position in the genome with genetic material ancestral to the sample, the number of eligible links *B*_*i*_ carried by each lineage *i* is therefore determined by the number of sites within the half-closed interval [*l*_*min*_, *r*_*max*_) [26]. The total rate at which lineage *i* recombines is therefore *rB*_*i*_. We can then compute the total recombination rate among lineages in population *k* as $r\sum_{i=1}^{a_{k}}B_{i}$.

**Migration events**: Lineages migrate between populations *k* and *l* at rate *γ*_*kl*_ in forwards time. The total rate at which all lineages in population *k* migrate to another population is therefore $a_{k}\sum_{l\neq k}^{q}\gamma_{kl}$.

As in other coalescent models, the time to the next event of each type is exponentially distributed according to the rate of each event type. Furthermore, we assume the coalescent, recombination and migration processes are independent conditional upon the number of lineages *a*_*k*_ in each population state. That is, while events may change the number of lineages in each state, the different events do not influence the probability of the other events occurring over time intervals in which *a*_*k*_ is constant. The three processes are therefore independent, competing processes where the time to the next event is exponentially distributed according to the total rate Ω at which events of any type occur:

Ω=∑k=1q((ak2)λk+r∑i=1akBi+ak∑l≠kqγkl). (1)

#### The likelihood of an ARG under the structured coalescent with known ancestral states

We now consider how to compute the likelihood of a fully known ancestral recombination graph G, where all events in the graph are observed including the source and destination of each migration event such that the ancestral location of all lineages is known at any point in time. Going backwards in time, at a coalescent event two lineages in state *k* merge into a single parent and the total number of lineages *a*_*k*_ in the ARG in state *k* decreases by one. At a recombination event, a lineage divides into two parent lineages and *a*_*k*_ increases by one. We seek to compute the likelihood L(G|θ) of G under the SCAR model given a set of demographic parameters *θ* = {λ, r, *γ*}, allowing for likelihood-based inference of these parameters from an ARG.

For an ARG with *e*_*c*_ coalescent events, *e*_*r*_ recombination events, and *e*_*m*_ migration events, there will be a total of *e* = *e*_*r*_ + *e*_*c*_ + *e*_*m*_ events in the graph. The ARG can be divided into *e* *tree intervals*, within which the total number of lineages in the ARG (and in each population) remains the same. We will let $a_{k}^{s}$ be the number of lineages present in the tree during the *s*-th tree interval. We denote the waiting time between each event as Δ*t*_*s*_ = *t*_*s*_ − *t*_*s*−1_.

In order to track which lineages are involved in particular events, let *h*(*s*) be a function that returns the lineage(s) involved in a particular event *s*. We then use the notation *w*_*h*(*s*)_ and *v*_*h*(*s*)_ to refer to the state of the lineages involved in event *s*, which is either a migration event from subpopulation *w*_*h*(*s*)_ to *v*_*h*(*s*)_, a coalescent event in population *v*_*h*(*s*)_ or a recombination event involving lineage *h*(*s*). Assuming exponentially distributed waiting times between events, the coalescent likelihood has the general form:

L(G|θ)=∏s=1e[exp(-∑k=1q[(aks2)λk+aks∑l≠kqγkl+r∑i=1aksBi]Δts)·(δemsγwh(s)vh(s)+δecsλvh(s)+δersrBh(s))] (2)

The exponential term gives the probability that in the *s*th time interval with duration Δ*t*_*s*_ no coalescent, recombination or migration event occurs in any population. The remaining term is the point probability density of the event that terminates the interval. We use the indicator variables $\delta_{e_{c}}^{s}$, $\delta_{e_{r}}^{s}$ and $\delta_{e_{m}}^{s}$ to indicate whether the event terminating interval *s* is a coalescent, migration or recombination event, respectively; where $\delta_{e_{•}}^{s}$ is 1 when the corresponding event type terminates the interval and 0 otherwise.

#### The likelihood of an ARG with unknown ancestral states

Because we typically do not observe the ancestral location or state of lineages, they must either be jointly inferred along with the other model parameters or integrated (marginalized) out when computing the likelihood of the ARG. Here, we use the approximation first proposed by Volz [21] to track the ancestral state of lineages probabilistically, and then marginalize over ancestral states using these lineage state probabilities.

With unknown ancestral states, the rate at which a pair of lineages *i* and *j* coalesce now depends on the probability that both lineages are in the same population at time *t* in the past:

$$\begin{array}{c} \lambda_{ij}\left(t\right)=\sum_{k}^{q}\frac{p_{ik}\left(t\right)p_{jk}\left(t\right)}{N_{k}}, \end{array}$$ (3)

where *p*_*ik*_ and *p*_*jk*_ are the probabilities that lineage *i* and lineage *j* are in state *k*, respectively. How these lineage state probabilities are computed is explained further below in *Tracking lineage state probabilities*.

The total rate at which all lineages *a* coalesce can then be computed by summing over all pairs of lineages:

$$\begin{array}{c} \lambda \left(t\right)=\sum_{i}^{a}\sum_{j\neq i}^{a}\sum_{k}^{q}\frac{p_{ik}\left(t\right)p_{jk}\left(t\right)}{N_{k}}. \end{array}$$ (4)

However, repeatedly summing over all possible pairs of lineages can become computationally burdensome, especially as the number of lineages grows large. To avoid this, we can approximate the number of lineages in each state using the lineage state probabilities:

$$\begin{array}{c} {\hat{a}}_{k}\left(t\right)=\sum_{i=1}^{a}p_{ik}\left(t\right). \end{array}$$ (5)

We then approximate the total rate at which pairs of lineages coalesce in state *k* as:

$$\begin{array}{c} \Lambda_{k}\left(t\right)=\text{max}[0,\frac{{\hat{a}}_{k}\left(t\right)\left({\hat{a}}_{k}\left(t\right)-1\right)}{2}]\frac{1}{N_{k}}. \end{array}$$ (6)

We then compute the total recombination rate in state *k* as:

$$\begin{array}{c} R_{k}\left(t\right)=r\sum_{i=1}^{a}B_{i}p_{ik}\left(t\right). \end{array}$$ (7)

The total likelihood of the ARG when integrating over ancestral states then becomes:

$$\begin{array}{c} L\left(G|\theta \right)=\prod_{s=1}^{e}[\text{exp}(-\sum_{k=1}^{q}[\Lambda_{k}\left(t_{s}\right)+R_{k}\left(t_{s}\right)]\Delta t_{s})\cdot (\delta_{e_{c}}^{s}\lambda_{v_{h(s)}}+\delta_{e_{r}}^{s}rB_{h(s)})] \end{array}$$ (8)

Note that while the migration rates do not directly enter into likelihood function they influence the lineage state probabilities *p*_*ik*_ that in turn determine Λ_*k*_ and *R*_*k*_.

The rates Λ_*k*_(*t*_*s*_) and *R*_*k*_(*t*_*s*_) are assumed to be piecewise constant between events in (8). If the waiting times Δ*t*_*s*_ between events are long such that these rates change significantly over a time interval, we can increase the numerical accuracy of the likelihood calculation by dividing each time interval *s* into *x*_*s*_ shorter sub-intervals:

$$\begin{array}{c} L\left(G|\theta \right)=\prod_{s=1}^{e}[\prod_{z=1}^{x_{s}}[\text{exp}(-\sum_{k=1}^{q}[\Lambda_{k}\left(t_{s,z}\right)+R_{k}\left(t_{s,z}\right)]\Delta t_{s,z})]\cdot (\delta_{e_{c}}^{s}\lambda_{v_{h(s)}}+\delta_{e_{r}}^{s}rB_{h(s)})], \end{array}$$ (9)

where Δ*t*_*s*, *z*_ is the length of sub-interval *z* between times *t*_*s*, *z*_ and *t*_*s*, *z*−1_.

#### Tracking lineage state probabilities

Going backwards in time, a lineage currently residing in population *k* will migrate to population *l* at rate *γ*_*lk*_. Assuming the probability of a lineage residing in a population is independent of the location of all other lineages, the migration process along each lineage can be modeled as a continuous time Markov process on a discrete state space [21]. We can then use a system of differential equations to track how the probability of a lineage residing in each state changes backwards through time:

$$\begin{array}{c} \frac{d}{dt}p_{ik}=\sum_{l}^{q}(p_{il}\gamma_{kl}-p_{ik}\gamma_{lk}). \end{array}$$ (10)

Given a vector of initial lineage state probabilities *p*_*i*_(0) at time zero, we can analytically solve (10) above for *p*_*i*_(*t*) at some time *t* further in the past:

$$\begin{array}{c} p_{i}\left(t\right)={\text{exp}}^{Qt}p_{i}\left(0\right), \end{array}$$ (11)

where the matrix *Q* is that transition rate matrix derived from *γ*:

$$Q=[\begin{array}{cccc} -\sum_{k}\gamma_{k,1} & \gamma_{1,2} & \cdots & \gamma_{1,q}\\ \gamma_{2,1} & -\sum_{k}\gamma_{k,2} & \cdots & \gamma_{2,q}\\ \vdots & \vdots & \ddots & \vdots \\ \gamma_{q,1} & \gamma_{q,2} & \cdots & -\sum_{k}\gamma_{k,q} \end{array}].$$

As originally shown in [23], these equations are approximate because they assume all lineages evolve independently such that the probability of one lineage residing in a population is completely independent. In contrast, under the exact structured coalescent model, lineages states may be correlated because the observation that two lineages have or have not coalesced can be informative about their location. For example, two or more lineages are unlikely to reside in the same population over long periods of time and not coalesce if *N*_*k*_ is small in population *k*, such that the observation that the lineages have not coalesced increases the probability of these lineages being in different populations. The bias introduced by ignoring the non-independence of lineages is most extreme when: 1) migration rates are low relative to coalescent rates and 2) either coalescent or sampling fractions are highly asymmetric between populations [23]. In these cases, a more accurate approximation to the structured coalescent exists but computing lineages state requires solving a high-dimensional system of differential equations. We therefore continue to assume independence among lineages but note that this more complex approximation can be substituted when necessary.

### Statistical inference under the SCAR model

We use a Bayesian MCMC approach to infer the posterior distribution of demographic parameters. In particular, we use a Metropolis-Hastings algorithm to sample from the joint posterior distribution of parameters given a fixed ARG G:

$$\begin{array}{c} p(\theta |G)\propto L(G|\theta )p(\theta ), \end{array}$$ (12)

where the likelihood L(G|θ) is computed as in (8) and *p*(*θ*) is the prior distribution on the demographic parameters. In simulation experiments, we chose a uniform distribution for *p*(*θ*) such that our estimates are minimally influenced by the prior but use informative priors when performing inference from real data.

### ARG reconstruction using ARGweaver

We use ARGweaver [12] to reconstruct ARGs from sampled genomic sequence data. ARGweaver uses the SMC approximation of McVean and Cardin [13] to compute the likelihood of an ARG evolving under the coalescent with recombination. In the model assumed by ARGweaver, exactly one recombination event is assumed to occur at each recombination breakpoint. A recombination event may not necessarily alter the topology of two neighboring trees in the ARG because a recombination event may only alter the time at which two lineages coalesce, but recombination events that affect neither the topology nor coalescent times are ignored. Coalescent events are further constrained to occur at discrete time points. The coalescent likelihood of the ARG is then combined with the likelihood of the sequence data evolving along each local tree in the ARG to compute the joint likelihood of the sequence data and ARG. ARGweaver then employs a Bayesian MCMC approach to sample ARGs from the corresponding posterior distribution. To obtain a single, representative ARG, we choose the ARG from the posterior sample with either the maximum joint likelihood, maximum (sequence) likelihood, or from the final MCMC iteration.

To facilitate computing the likelihood of ARGs under the SCAR model, we convert the ARG obtained from ARGweaver to the tskit tree sequence format [26]. The tskit tree sequence format provides a concise encoding of an ARG as a series of correlated local trees corresponding to the genealogy of the sample over different genomic regions [26, 31]. The tree sequence format also facilitates computing the likelihood of the ARG under the SCAR model. We can simply perform a post-order traversal through the ARG by iterating over each node, computing the likelihood of the event at the node, updating the edges (i.e. lineages) present in the ARG after the event, and computing the likelihood of no event occurring between nodes as in Eq (8). Code for converting ARGs into tskit tree sequence format and computing the likelihood of the ARG is available at https://github.com/sunnyfangfangguo/SCAR_project_repo.

### Simulation study

We simulated ARGs along with genomic sequence data to test the accuracy of ARG reconstruction using ARGweaver and the statistical performance of inference under the SCAR model before applying the method to real data. Simulated ARGs (tree sequences) were generated by msprime [26]. To test the accuracy of ARGweaver in reconstructing ARGs, sequence alignments for each local tree in an ARG were generated with a HKY substitution model [32] with the transition/transversion ratio *κ* = 2.75 using Pyvolve [33]. Our simulations are similar to those of [12], which assumed a fixed effective population size *N*_*e*_ = 100, genome length *L* = 10,000, and recombination rate per site per generation *r* = 2.5*e* − 06, and varied the mutation-to-recombination rate ratio *μ*/*r* from 1 to 2048. 100 simulations were conducted for each *μ*/*r* ratio.

In order to quantify ARG reconstruction accuracy, normalized Robinson-Foulds (RF) distances [34, 35] between corresponding simulated local trees and inferred local trees for each genome region were calculated as a metric of local tree accuracy, which varies between 0 and 1. Kendall-Colijn (KC) distances, which in addition to tree topology also consider differences in branch lengths, were also computed between simulated and inferred local trees [36]. RF distances and KC distances along the whole chromosome were then calculated as an average distance over all genome regions. We also compared the true number of recombination events in the simulations to the number of recombination events inferred by ARGweaver. From S2 Fig, we can clearly see that the ARG with the maximum iteration included the number of recombination events closest to the true number, while ARGs with the maximum likelihood consistently overestimated and ARGs with the maximum joint likelihood consistently underestimated the number of recombination events across all the ratios. Thus, we selected the ARG with the maximum iteration to show the accuracy of ARGweaver.

In order to test demographic inference under the SCAR model, three simulation experiments were run: we (1) estimate the effective population size, recombination rate and migration rate directly from the true simulated ARG; (2) jointly estimate the recombination rate and migration rate from the true ARG; and (3) estimate the effective population size, recombination rate, and migration rate from ARGs inferred by ARGweaver. When estimating migration rates between populations, we treat the ancestral location of each lineage as unknown and track the state of each lineage probabilistically. Again, 100 simulations were run for each simulation experiment. In the first two experiments, for each simulation, the true value of the estimated parameter(s) were drawn from an evenly spaced grid of values, while other parameters were kept constant. When only estimating the effective population size or recombination rate, we simulate ARGs without population structure.

## Results

### Testing the accuracy of ARG inference using ARGweaver

#### Accuracy of local trees in ARGweaver inferred ARGs

**Fig 2:**
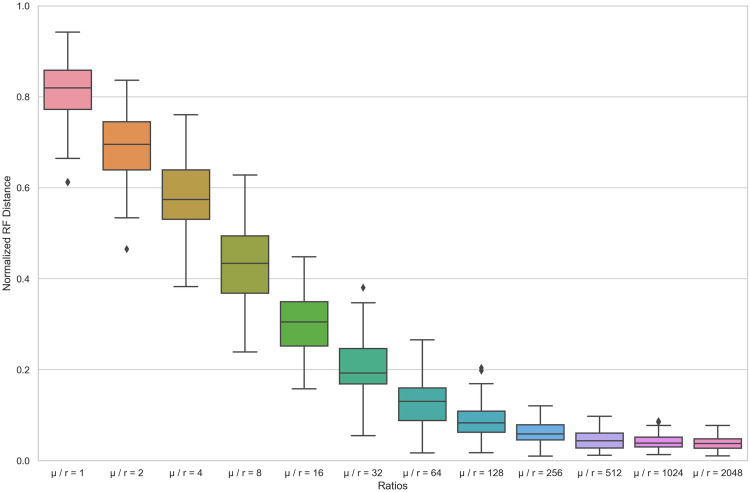
Normalized RF distances between the true (simulated) local trees and the local tree inferred by ARGweaver in the reconstructed ARG under different ratios of mutation rate / recombination rate. For each simulation, *N*_*e*_ is 100, sample size is 50, genome length is 1e04, and recombination rate *r* is 2.5e-06. Under each ratio, 100 simulations were run.

Because our inference methods ultimately rely on the ability to accurately reconstruct ARGs, we first test the ability of ARGweaver to reconstruct ARGs from genomic data simulated under different mutation-to-recombination rate ratios *μ*/*r* in order to vary the number of phylogenetically informative sites (SNPs) between each recombination breakpoint. To evaluate the accuracy of ARGweaver, we use normalized Robinson-Foulds (RF) distances to quantify the topological differences between the simulated and reconstructed local trees in the ARG. From Fig 2 we can see that, with increasing *μ*/*r* ratios, the median RF distances decrease from 0.819 to 0.037, showing a clear increase in ARGweaver’s performance to accurately reconstruct the topology of local trees in the ARG. Likewise, Kendall-Colijn (KC) distances, which take into account branch lengths in addition to tree topology, show a similar trend of improved performance with increasing *μ*/*r* ratios (S3 Fig). This trend is likely due to the fact that sequence diversity, and thus the number of phylogenetically informative sites between each recombination breakpoint, increases with the *μ*/*r* ratio (S4 Fig).

#### Accuracy in the number of inferred recombination events

**Fig 3:**
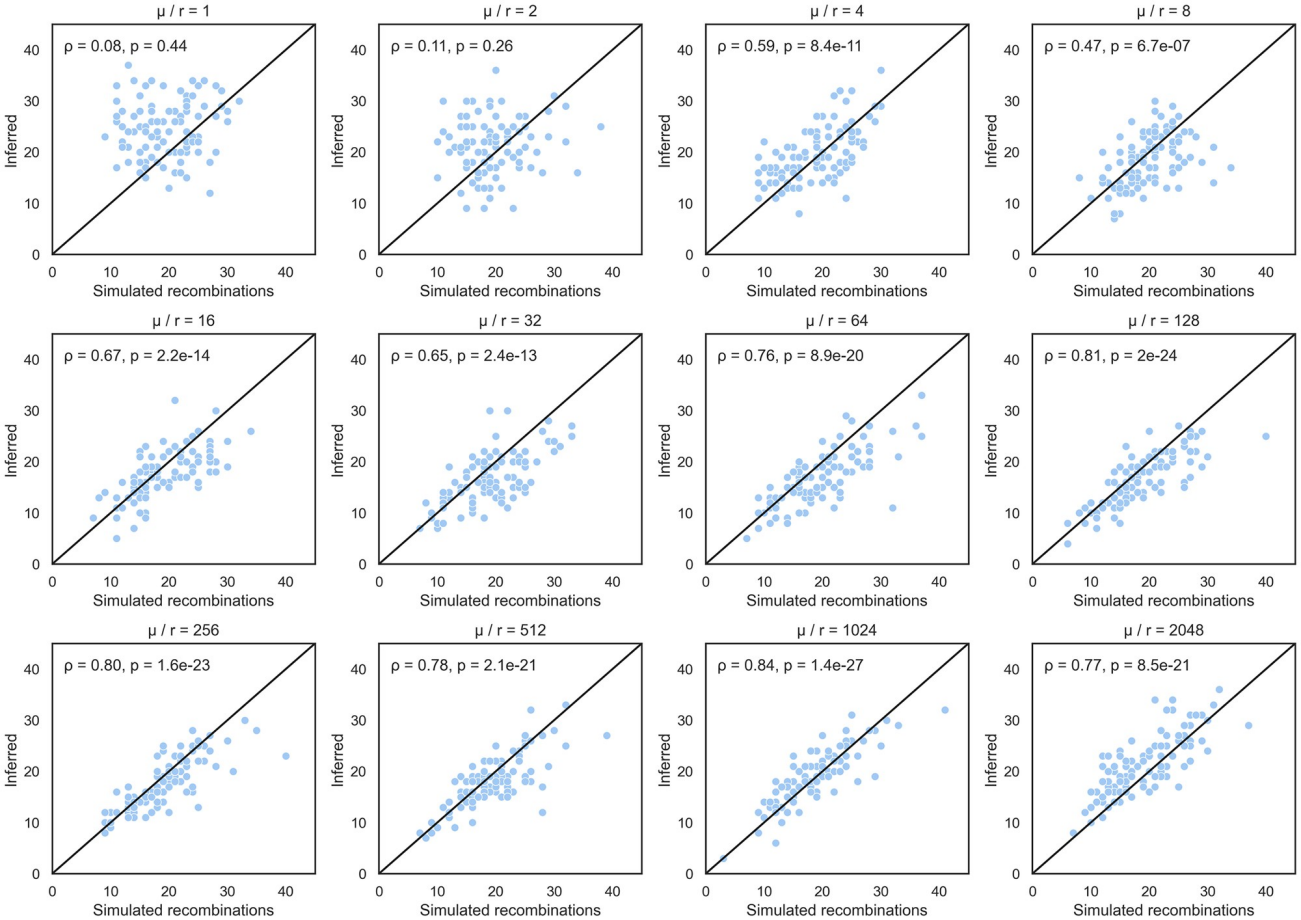
Simulated number of recombination events versus number inferred by ARGweaver under different ratios of mutation rate / recombination rate. Each diagonal line is *x* = *y*. *ρ* and *p* are the correlation coefficient between the simulated and estimated number of events and corresponding p-value, respectively. We selected the ARG sampled by ARGweaver during the final MCMC iteration to count the number of recombination events.

We compared the number of recombination events inferred by ARGweaver against the true number known from simulations under 12 different *μ*/*r* ratios to further test the accuracy of ARGweaver. The number of recombination events inferred by ARGweaver was significantly and positively correlated with the true number of recombination events when the *μ*/*r* ratio ≥ 4, while at lower ratios the correlation is poor indicating it may not be possible to estimate the true number of recombination events unless the mutation rate is at least several times higher than the recombination rate (Fig 3). As the *μ*/*r* ratio increases, the correlation generally becomes stronger.

### Testing the SCAR model on simulated ARGs

#### Statistical performance of estimating *N*_*e*_, recombination rate *r*, and migration rate *M*

**Fig 4:**
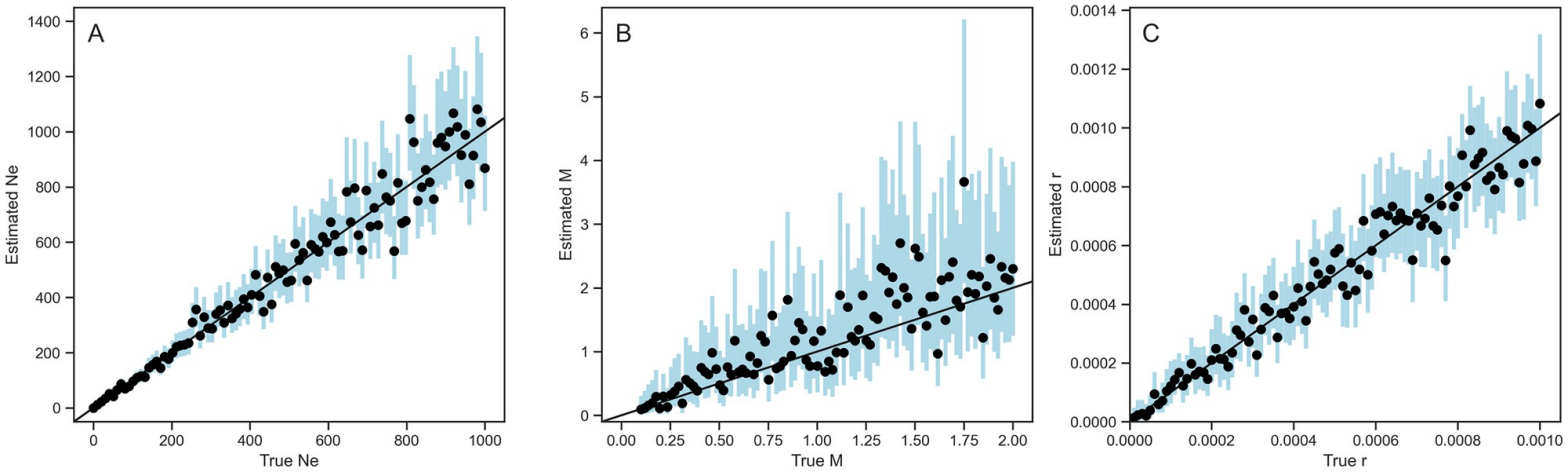
Inference of effective population size *N*_*e*_ (A), migration rate *M* (B), and recombination rate *r* (C) from 100 known ARGs. Migrations rates are assumed to be equal (symmetric) between two populations. Dots and blue bars represent the median posterior estimates and the 95% credible intervals for each simulation.

Next, we tested how well we are able to estimate effective population sizes *N*_*e*_, recombination rates *r*, and migration rates *M* from simulated ARGs known without error under the SCAR model. Fig 4 shows that the SCAR model can accurately estimate all three of these parameters across a wide range of true values. Table 1 summarizes the performance of our estimates across simulations in terms of the relative bias, coverage of the 95% credible intervals, and calibration between true and estimated parameters. We find that migration rate estimates are very accurate when the true migration rates are smaller than 1 per unit time. However, in some simulations the migration rates are overestimated, especially when the true rate was larger than 1, indicating an inability to precisely estimate high rates likely due to the fact that the likelihood function becomes very flat across a wide range of higher rates. After testing the SCAR model on simulations with different sample sizes (S5 Fig), we found that the additional information provided by increased sampling could provide more accurate and precise migration rate estimates.

**Table 1:** The relative bias, coverage, and calibration of estimating *N*_*e*_, *r*, *M* by the SCAR model.

Parameter	Relative error	Coverage	Calibration
*N*_*e*_	+1.23%	92 in 100 times	0.98
*r*	+1.87%	97 in 100 times	0.98
*M*	+22.36%	95 in 100 times	0.85

Except for the parameter being systematically varied, all parameters were fixed at constant values: effective population sizes *Ne* = 1.0, sample sizes *k* = 100, genome length *L* = 10000, recombination rate *r* = 0.0, migration rate *M* = 0. For models with migration, migration rates are assumed to be symmetric between two subpopulations

**Fig 5:**
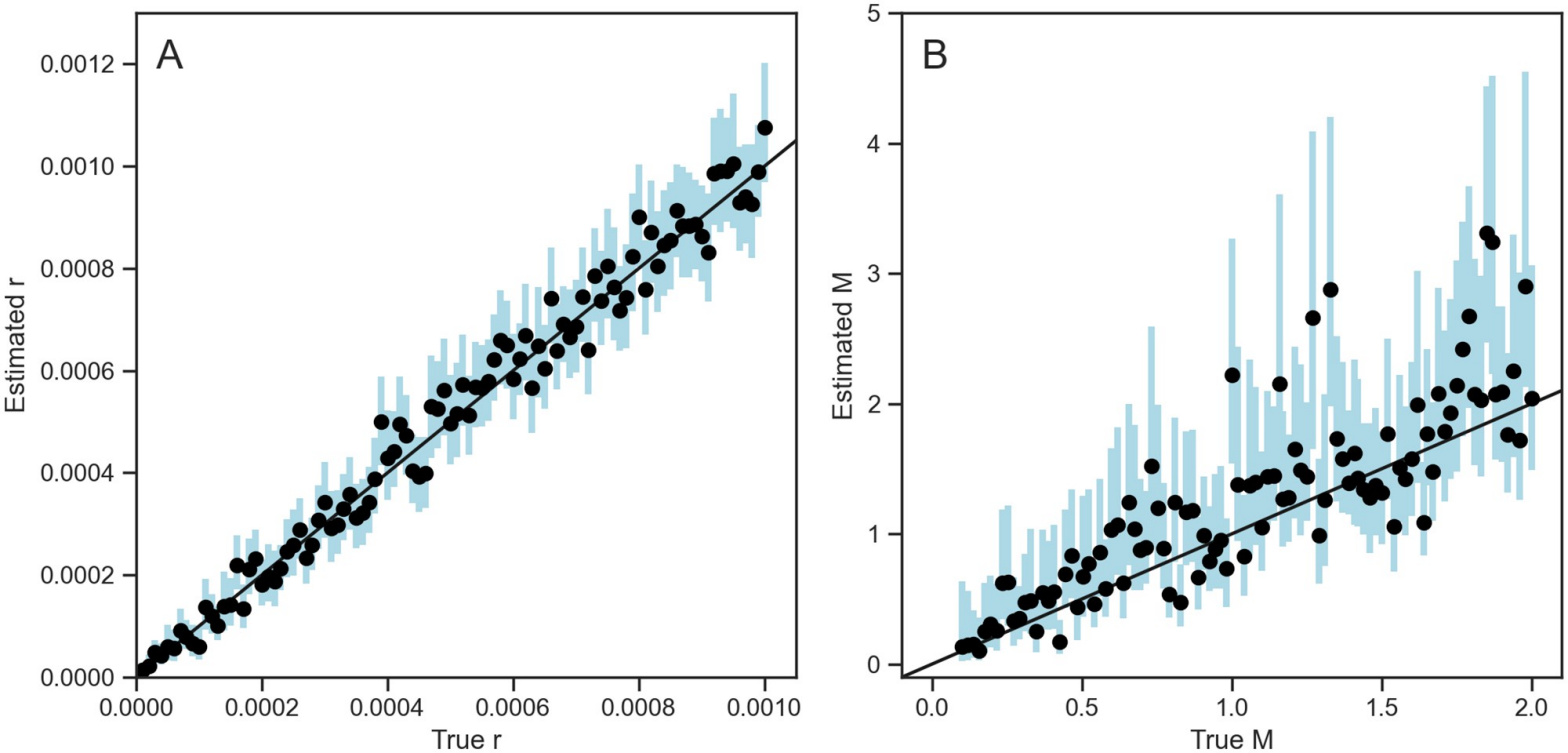
Joint estimation of recombination and migration rates together. Dots and blue bars represent the median posterior estimates and the 95% credible intervals of the marginal posterior distribution of each parameter from each simulation.

We further tested the performance of the SCAR model when jointly estimating the recombination rate *r* and migration rate *M* together. As shown in Fig 5, the SCAR model can accurately infer the marginal posterior distribution of each parameter even when the two parameters are jointly estimated together.

#### Statistical performance of estimating recombination rates from ARGs inferred by ARGweaver

**Fig 6:**
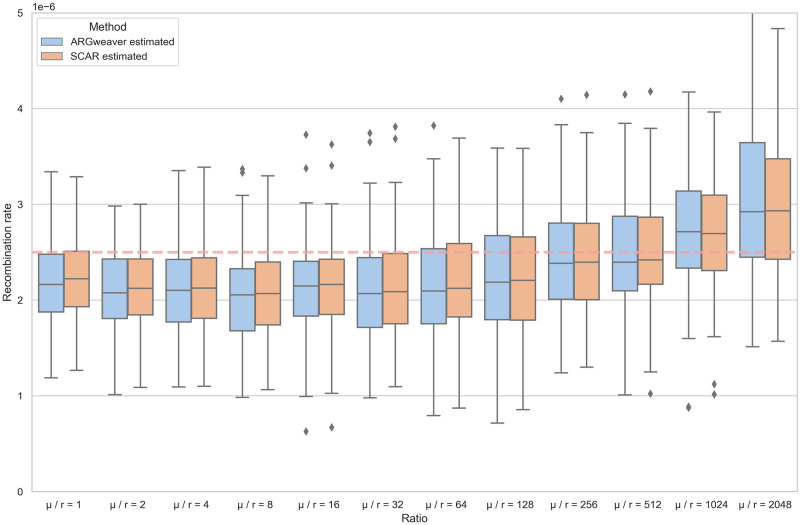
Recombination rates estimated using ARGweaver and SCAR from ARGs inferred by ARGweaver under different *μ*/*r* ratios. The dashed red line is the simulated recombination rate in all simulations. Under each *μ*/*r* ratio, 100 simulations were run.

In order to see how ARG reconstruction errors influence our estimates, we estimated recombination rates using the SCAR model from ARGs reconstructed by ARGweaver rather than the true simulated ARGs. We also compared to the recombination rates estimated by ARGweaver, which simply counts recombination events in the reconstructed ARG and divides by the total branch-length of the ARG to estimate the recombination rate [37]. From Fig 6 we can see that the accuracy of recombination rates estimated by both SCAR and ARGweaver improves with increasing *μ*/*r* ratios. However, when the *μ*/*r* ratio exceeds 1024, recombination rates become slightly over-estimated. We suspect that the increased accuracy of recombination rate estimates at higher *μ*/*r* ratios is due to increasing phylogenetic information about the local trees and thus power to distinguish true recombination events from uncertainty in the topology of local trees. However, at very large ratios individual sites may become phylogenetically uninformative due to recurrent or convergent mutations (i.e. saturation effects) and we therefore may become overconfident that discordance between local trees is due to recombination rather than phylogenetic errors.

Finally, we tested the performance of jointly estimating effective population sizes, recombination and migration rates when the ARG was simulated under a structured two-deme model but reconstructed assuming a single panmictic population model in ARGweaver. Despite this model misspecification, parameter estimation is accurate and generally improves with increasing *μ*/*r* ratios. However, *N*_*e*_ was slightly underestimated and migration rates overestimated even at very high *μ*/*r* ratios (S6 Fig). These small biases likely result from ARGweaver systematically under-estimating branch lengths and coalescent times under a misspecified coalescent model ignoring population structure. However, these results more generally suggest that the coalescent prior assumed when reconstructing the ARG has very minimal impact on downstream demographic inference from the ARG.

### Recombination and migration in *Aspergillus flavus*

It is estimated that over 25% of food crops are contaminated with mycotoxins worldwide [38]. *Aspergillus flavus*, a pathogen of plants and animals, is a major aflatoxin producer that has a broad economic impact [39]. *A. flavus* can infect and contaminate preharvest and postharvest seed crops with the carcinogenic secondary metabolite aflatoxin [40]. This fungus is predominantly haploid and homokaryotic [41]. *A. flavus* was thought to be cosmopolitan and clonal, until evidence for genetic recombination due to a cryptic sexual state were reported [42] and later the sexual stage was described [43]. In natural populations, *A. flavus* undergoes both sexual and asexual reproduction [44, 45]. Previous studies also found extensive recombination in the ancestral history of the aflatoxin cluster [46, 47], which is a 70-Kb-gene-cluster near chromosome 3’s right telomeric region [40, 48]. Based on multilocus DNA sequence markers in the aflatoxin cluster (*aflM/aflN* and *aflW/aflX*) and three other nuclear loci (*mfs, amdS, trpC*), this fungus can be delimited into two evolutionary distinct lineages: IB and IC, where IB includes mainly nonaflatoxigenic isolates while IC includes both toxigenic and atoxigenic strains [46, 49]. There is evidence that *A. flavus* has the potential for long-distance dispersal via conidia [50–52], but movement between geographic locations is poorly characterized.

Given that both recombination and migration shape the evolutionary history of *A. flavus*, here we aim to use ARGweaver and our SCAR model to explore the two evolutionary forces together by reconstructing ARGs, and then estimating the recombination and migration rates.

The genome size of *A. flavus* is about 37 Mb on eight chromosomes [53], but we focused our analysis on the migration and recombination history of chromosome 3, which is about 5 Mb. A total of 51 lineage IB strains and 48 lineage IC strains were collected across the United States, including Arkansas, Indiana, North Carolina, and Texas in 2013 (Table 2) [54]. Sample metadata is provided in supporting information S1 Table. Single-nucleotide polymorphism (SNP) genotyping was performed across chromosome 3 with *A. oryzae* RIB40 as the reference genome [55]. Because there was limited migration between lineages IB and IC [54], we analyzed the two lineages separately. No SNPs in the aflatoxin gene cluster were included for IB because few isolates harbored this gene cluster.

**Table 2:** Sampling locations and numbers for lineages IB and IC.

State	Samples location	Lineage IB	Lineage IC
Arkansas	Newport Research Station; 35.57°N, 91.26°W	11	17
Indiana	Southeast-Purdue Agricultural Center; 39.03°N, 85.53°W	3	13
North Carolina	Upper Coastal Plain Research Station; 35.89°N, 77.68°W	11	13
Texas	Texas A & M University Farm; 30.55°N, 96.43°W	26	5

We used ARGweaver to infer ARGs from SNPs spanning most of chromosome 3. Because ARGweaver requires an estimate of the recombination rate to infer ARGs, we used LDhat version 2.2 [56] to estimate Watterson’s theta and the population recombination rate. SNP data were filtered using a series of different missing data thresholds before running LDhat, and we estimated the median recombination rate across filtered data sets. The *A. flavus* mutation rate was previously estimated as 4.2e-11 per site per mitosis [57], which can be converted to 2.82e-09 per base per generation. Given this mutation rate, the effective population size *N*_*e*_ was calculated from Watterson’s theta (88.23 and 559.04 for lineages IB and IC, respectively). ARGweaver also needs a maximum time threshold for coalescent events, which was set as the expected time to the most recent common ancestor based on the sample size and estimated population sizes. With these parameters, we ran ARGweaver for 20,000 iterations with 1000 iterations as burn-in. To keep ARGweaver’s run time manageable, we compressed blocks of 5 variable sites by conditioning the breakpoints between each block in a flexible manner so that no more than one variant site in the same block was chosen [37]. All the runtime parameters can be found in the supporting information S2 Table. From the SMC files produced in the iterations, we choose the ARG with the maximum joint-likelihood as our best estimate of recombination patterns across chromosome 3.

We used a tanglegram to show the topological changes between neighboring local trees in the inferred ARG. In a tanglegram, each local tree is drawn, and then auxiliary lines are drawn to connect matching taxa in neighboring trees. If there is no recombination, the lines connecting matching taxa should be horizontal whereas crossing lines can be used as a visual heuristic to assess the extent of recombination. We use the python package baltic [58] to display the tanglegrams (S7 Fig).

The ARGs reconstructed in ARGweaver were then used to estimate recombination and migration rates using our SCAR model. For these analyses we used exponential priors (for IB *r* ∼ *Exp*(1.37*e* − 09), *m*_*ij*_ ∼ *Exp*(0.1); for IC *r* ∼ *Exp*(2.17*e* − 10), *m*_*ij*_ ∼ *Exp*(0.1)). MCMC chains were run for 40,000 iterations. Besides estimating these parameters from the reconstructed ARGs, we also compared the posterior distributions of estimated migration rates from the consensus tree of all the local trees in the ARG along the whole chromosome using the SCAR model. Additionally, we compared how the RF distances between pairs of local trees for different regions of the genome changed based on their genomic distance, which was the absolute value of coordinates (middle of genome segment location) of the difference between two trees.

#### The reconstructed ARGs for chromosome 3 of lineages IB and IC

The *μ*/*r* ratios were calculated using Watterson’s *θ* and the population recombination rate obtained by LDhat. For lineage IC, the *μ*/*r* ratio of the entire chromosome 3 was 13, whereas for lineage IB *μ*/*r* was 2.05. Even though the *μ*/*r* ratio of lineage IB was slightly lower than the lower limit at which we found ARGweaver could accurately reconstruct ARGs in simulations, we continued with the analysis in order to explore the limits of ARG-based phylogeographic inference.

We reconstructed ARGs for chromosome 3 of the *A. flavus* genome from 51 lineage IB isolates and 48 lineage IC isolates. Overall, the ARGs contained 190 recombination events for lineage IB and 774 recombination events for lineage IC. To visualize how the topology of local trees varied across the genome, we plotted tanglegrams for the first 10 local trees in each ARG for lineage IB (S7A Fig) and lineage IC (S7B Fig), as well as the 12 local trees in the aflatoxin gene cluster for lineage IC (S7C Fig). Although there was always one recombination event between each local tree in the ARG reconstructed by ARGweaver, not all recombination events result in topological discordance between neighboring trees. In the ARG of lineage IB, 92.1% of recombination events caused topological discordance between local trees whereas the other 7.9% only changed coalescent times. In the ARG of lineage IC, 98.3% of recombination events resulted in topological discordance while only 1.7% caused changes in coalescent times. The average RF distance between neighboring local trees for lineages IB and IC was 9.23 and 10.57, respectively; and the average normalized RF distance between local trees for IB and IC was 0.094 and 0.115, respectively; whereas the average effect of a single random SPR move on the IB and IC local trees resulted in an RF distance of 17.93 and 18.19, respectively (S8 Fig). Thus, while there can be considerable phylogenetic discordance between local trees, recombination events tend to be more topologically conservative and occur between more closely related lineages than would be expected by chance from truly random SPR moves. Moreover, some phylogenetic discordance may be due to errors in reconstructing local trees, especially because the distance between pairs of breakpoints along the chromosome were often relatively small (S9A Fig), such that many non-recombining segments likely did not have enough segregating sites to reconstruct local tree accurately. Overall though, we found that the normalized RF distance between pairs of local trees increased logarithmically with their distance from each other in the genome (S9B Fig), consistent with discordance being driven by recombination rather than phylogenetic error over larger genomic distances.

**Fig 7:**
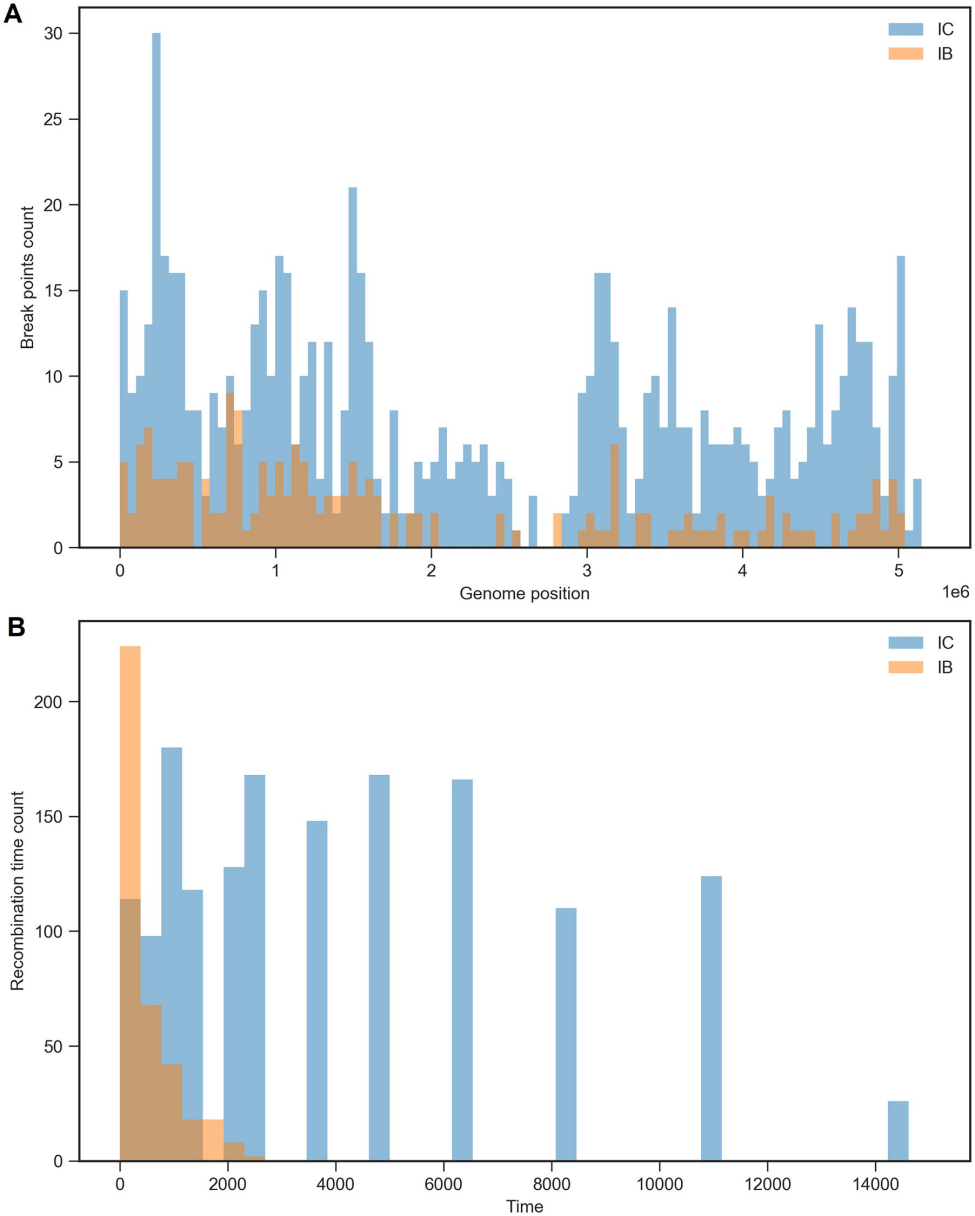
Inferred recombination events in *A. flavus* chromosome 3 (A) and the frequency of recombination time (generations in the past)(B), respectively, of lineages IB and IC.

Recombination breakpoints were distributed unevenly across the genome, with the putative centromeric region containing far fewer recombination events for both lineages (Fig 7A). Fig 7B shows the distribution of recombination times for both lineages. While recombination events occurred mostly in the recent past for lineage IB, many recombination events occurred in the much deeper past for lineage IC.

#### Recombination rates of lineages IB and IC

**Fig 8:**
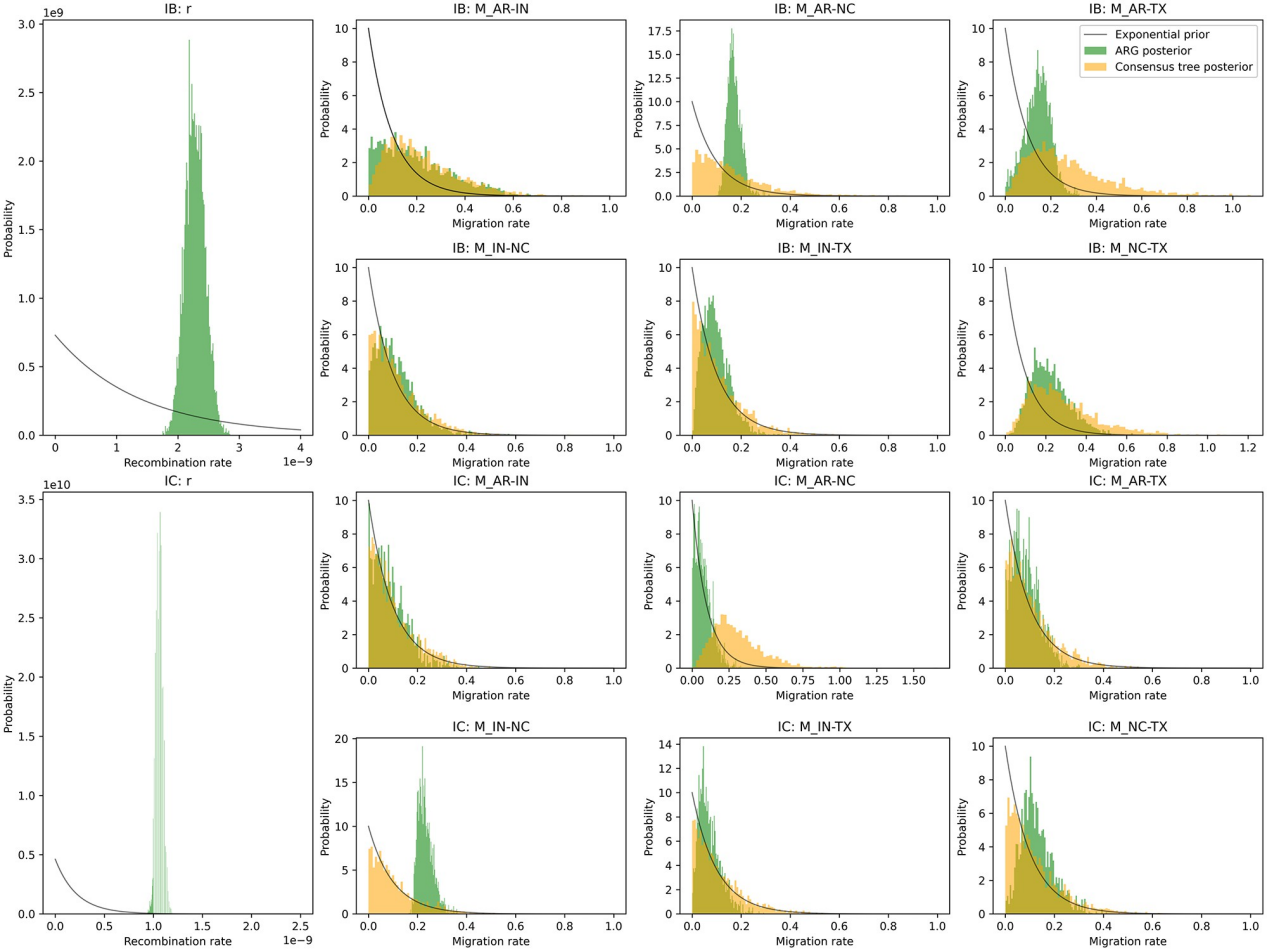
The prior and posterior distributions of demographic parameters estimated from either the full ARG or a single consensus tree for lineages IB and IC. For lineage IB, the mean value of exponential prior of recombination rate is 1.37e-09, and the mean value of exponential prior of migration rates is 0.1; For lineage IC, the mean value of exponential prior of recombination rate is 2.17e-10, and the mean value of exponential prior of migration rates is 0.1. Note that we cannot estimate *r* from the consensus tree so only the posterior distribution estimated from the ARG is shown.

Using the SCAR model, we estimated the recombination rate for lineages IB and IC (first column of Fig 8). The recombination rate of lineage IB was estimated to be 2.28E-09 per site per generation, with a *μ*/*r* ratio of 1.24. Here we assume the recombination rate is constant across both lineages and all of chromosome 3. The recombination rate of lineage IC was estimated to be 1.06E-09 per site per generation, with a *μ*/*r* ratio of 2.66. Although fewer recombination events were identified for lineage IB than lineage IC, lineage IB was estimated to have a higher recombination rate. This counter-intuitive result can be explained by the fact that lineage IB also has a smaller effective population size and thus coalescent times occur in the more recent past, resulting in less time for recombination events to occur in IB than in IC, consistent with the temporal distribution of recombination events observed in Fig 7B.

#### Migration rates of lineages IB and IC between subpopulations

**Fig 9:**
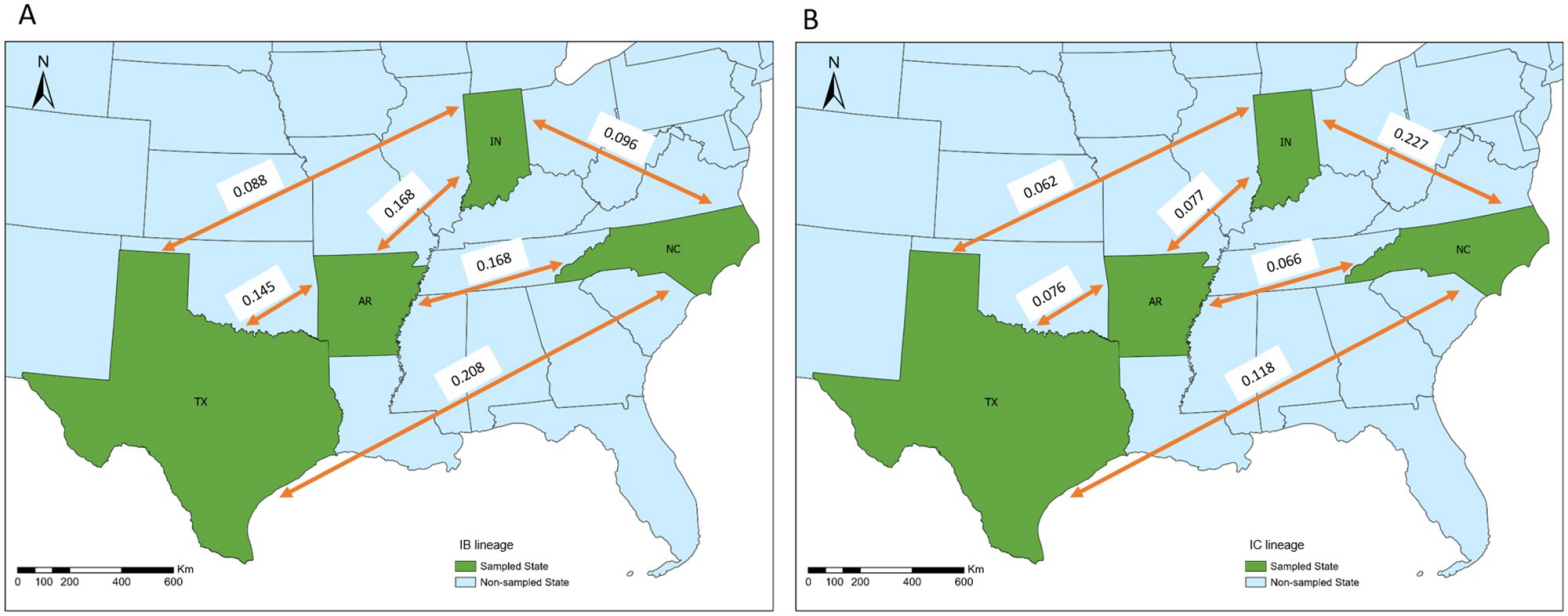
The migration rates (per generation) estimated for lineages IB (A) and IC (B) between subpopulations in four states. The base layer shapefile was downloaded from the website of United States Census Bureau https://www2.census.gov/geo/tiger/TIGER2019/STATE/.

Using the SCAR model, we estimated the migration rates of lineages IB and IC between subpopulations along with their recombination rate from their ARGs (Fig 8). Migration rates between subpopulations were found to vary between 0.05 and 0.2 migrations per generation, suggestive of extensive movement between populations. Migration rates between subpopulations were similar within each lineage (Fig 9). However, for lineage IB, the migration rate between subpopulations in North Carolina and Texas was slightly higher, and the Arkansas subpopulation had the highest migration rates to other subpopulations overall; for lineage IC, the migration rate between subpopulations in Indiana and North Carolina was slightly higher, as well as the migration rate between subpopulations in Texas and North Carolina. Generally, the migration rates of lineage IC were lower than for lineage IB.

We also compared migration rates estimated from full ARGs against migration rates estimated from a single phylogeny, in this case the consensus tree of each ARG. The posterior distribution of migration rates inferred from both the full ARG and the consensus tree are compared in Fig 8 and provided in supporting information S3 Table. Overall, the posterior distributions of migration rates estimated from the consensus tree diverged little from the prior distribution, indicating that the consensus trees contained little information about migration patterns. By contrast, the posterior distributions estimated from the full ARG were typically peaked with a much greater probability density concentrated around the posterior median relative to the prior distribution. These results suggest that we can obtain much more information from the full ARG than from any individual consensus tree (or gene tree), owing to the greater number of ancestral lineages and their associated migration histories in the ARG.

## Discussion

Because population structure and recombination jointly shape the genealogical history of many organisms, we developed SCAR to extend the structured coalescent to include ancestral recombination. When used for demographic inference, we showed that SCAR can successfully estimate effective population sizes, migration rates and recombination rates from reconstructed ARGs. We then showed that SCAR can recover these parameters accurately both from the true (simulated) ARGs and from ARGs reconstructed from genomic sequence data using ARGweaver, although performance declines as the recombination rate approaches the mutation rate. We also applied the SCAR model to *A. flavus* genomic data using ARGs inferred by ARGweaver, demonstrating how these methods can be applied to real world pathogens with complex histories of both migration and recombination.

While new methods for ARG reconstruction are being developed at a rapid pace, we chose ARGweaver as a gold-standard for inference as it reconstructs ARGs by sampling them from their full posterior distribution up to the approximation introduced by the Sequential Markov Coalescent [12, 13], which is known to be a very good approximation to the full coalescent with recombination [59]. Indeed, a recent simulation study found that ARGweaver was substantially more accurate in estimating coalescent times than other ARG reconstruction methods [60]. Other, more approximate methods may therefore be faster or allow larger samples sizes but are unlikely to outperform ARGweaver in terms of accuracy. We therefore used ARGweaver to explore the limits of reconstructing ARGs and estimating recombination rates from simulated data. Regardless of method, the number of phylogenetically informative sites (i.e., SNPs) between recombination breakpoints is likely the ultimate factor limiting accurate reconstruction of local tree topologies within an ARG and thereby our ability to distinguish true recombination events from topological discordance introduced by phylogenetic uncertainty. We therefore explored the limits of accurate ARG reconstruction by varying the ratio of the mutation rate to the recombination rate *μ*/*r*. We found that at high *μ*/*r* ratios, ARGweaver does in fact reconstruct ARGs very accurately. However, our ability to reconstruct local trees within the ARG rapidly degrades at lower *μ*/*r* ratios and as expected, our ability to accurately estimate recombination rates likewise decreases with our ability to accurately reconstruct ARGs. Our simulations suggest that a *μ*/*r* ratio of about 4 poses a practical lower limit on our ability to reconstruct ARGs. While many rapidly evolving viruses and predominately clonal bacteria exceed this threshold [61, 62], this definitely poses a challenge to accurate ARG reconstruction for many highly recombining bacteria and eukaryotic organisms. For example, *μ*/*r* ratios for fungi have been reported as low as 0.1 in *Zymoseptoria triciti* [63] and as high as 45.9 for *Glomus etunicatum* [64]. However, because recombination requires direct physical interactions (e.g. sexual reproduction), recombination rates can vary substantially even between populations of the same species based on the frequency at which individuals encounter one another [4, 5]. This suggests that ARG reconstruction methods will likely need to be applied on a one-by-one basis to particular data sets rather than being applied or dismissed for broad classes of organisms.

The SCAR model tracks the movement of lineages in an ARG between subpopulations by approximating ancestral state probabilities. Rather than jointly estimating the ancestral states with the other demographic parameters, we probabilistically track the movement of lineages and integrate over their unknown states using the approach first developed by Volz [21]. Using this method, we can accurately and quickly estimate migration rates from simulated ARGs. However, we found that SCAR overestimates migration events to some extent, especially when the true migration rates approach one per generation. This bias might be caused by assuming lineage state probabilities evolve independently across lineages and are independent of the coalescent process [21]. However, Müller et al. [23] showed that the lineage independence assumption performs worst when migrations rates are very low relative to coalescent rates (the opposite of our situation) and when coalescent rates are highly asymmetric between populations (a situation we do not consider). We therefore think it more likely that, for larger migration rates, there simply is not enough information to determine the true rate, as the likelihood surface remains essentially flat across a wide range of higher values (S10 Fig). Based on the results of estimating migration rates using different sample sizes, we show that the larger the sample size, the more accurate estimation becomes, verifying our speculation that biases in estimating higher migration rates were attributable to a lack of information.

Several earlier approaches likewise aimed to extend the structured coalescent to include recombination, including LAMARC 2.0 [65], CSD ${\hat{\pi }}_{\Theta }$ [66], ARGweaver-D [67], and SCoRe [68]. LAMARC 2.0 can simultaneously estimate migration rates, population growth rates, and recombination rates [65]. SCAR and LAMARC 2.0 model the recombination process in the same way [29], but like other early implementations of the structured coalescent [17], it uses MCMC to sample migration histories, limiting its applicability to larger data sets or data sets with more than a few sampled populations [69]. ARGweaver-D [67] extends ARGweaver to allow for demographic inference in structured populations under a user defined model. However, in ARGweaver-D migration events need to be fully specified in terms of their time, source, and recipient population; whereas SCAR allows for migration histories to be inferred from ARGs with no prior knowledge about individual migration events. Finally, SCoRe [68] can infer migration rates and reassortment patterns for segmented viruses from a phylogenetic network jointly estimated in BEAST2 [70]. Conceptually, SCoRe is very similar to SCAR in that both methods track the movement of lineages probabilistically based on similar approximations to the structured coalescent, although SCoRe uses more refined approximations to track lineage movement than what are currently implemented in SCAR. The main difference between SCAR and SCoRe is that the SCoRe model specifically focuses on reassortment, where different segments of a viral genome are inherited from different parents, leading to a block-like haplotype structure where all sites in the same segment necessarily share the same phylogenetic history. In contrast, SCAR allows for a more general model of recombination where recombination breakpoints and thus changes in local tree topologies can occur anywhere across the genome, leading to much more complex ARGs. The SCAR model therefore accommodates varying mechanisms of recombination, such as crossovers and gene conversion, and is thus applicable to a broader range of viral, bacterial, and fungal genomes.

For organisms like *A. flavus* that recombine frequently relative to their mutation rate, there may be further challenges to inferring ARGs given a low *μ*/*r* ratio. Furthermore, the aflatoxin gene cluster is reported to be a recombination hot spot [46], so the *μ*/*r* ratios likely vary across the genome [71], but we assume a constant mutation rate and recombination rate when reconstructing ARGs. While there are likely regions where recombination rates are lower and we can accurately reconstruct phylogenetic relationships, this will not be the case across the entire genome. Despite the inherent variability in ARG reconstruction accuracy across the genome, our estimates using ARGweaver/SCAR are consistent with those reported in the *A. flavus* literature. We found that the ratio of the mutation rate to recombination rate of lineages IB and IC was 1.24 and 2.66, respectively. In Drott et al. [72], the ratio of mutation rate to recombination rate in three populations calculated by ClonalframeML vary from 2.26 to 5.41. Even though our calculations were based on a single chromosome, they were similar in magnitude to genome-wide estimates for lineage IC. Moreover, we found that the putative centromeric region of the chromosome contains far fewer recombination events for both lineages IB and IC, which accords with the knowledge that regions surrounding centromeres are a cold spot of recombination [4, 73].

While incorporating recombination into phylogeography has typically been viewed as burdensome, considering recombination and the full ancestry of sampled genomes through an ARG allows us to track the ancestral movement of many different genes or genomic regions. Considering the full ARG rather than just a single phylogeny therefore provides more information about demographic parameters and allows us to see how migration histories vary across the genome. While it has long been appreciated that considering multiple ancestral histories across the genome can improve demographic inference [8, 74, 75], here we demonstrate that reconstructing ARGs for *A. flavus* provides much more information about migration between populations than does a single (consensus) tree. Indeed, posterior distributions for the *A. flavus* migration rates inferred from ARGs are concentrated around their posterior median while the same migration rates inferred from a single tree diverge little from the prior, demonstrating that it may be possible to estimate migration rates from ARGs even when a single tree contains no information about these parameters.

Using the SCAR model, we can now conduct phylogeographic inference using all the information contained within an ARG. In the future, we plan to combine the SCAR model with more computationally efficient methods for reconstructing ARGs like Espalier [76]. Rather than assuming a single panmictic population model when reconstructing the ARG, this would allow for the ARG and demographic parameters to be jointly inferred under a more flexible class of structured coalescent models. Because there can be considerable uncertainty surrounding ARG reconstructions, especially for populations with high recombination rates relative to mutation rates, we also plan to extend SCAR to marginalize demographic inferences over a set of sampled ARGs. Together, these advances will allow us to explore how recombination and migration jointly shape the phylogeographic history of a broad range of pathogens and other recombining organisms.

## Supporting information

S1 Fig One possible ARG of two samples resulting from the coalescent with recombination. Time starts at present (bottom) and increases going backward in time (top). The genome of each lineage is represented by a rectangle with blue filled regions containing material ancestral to the sample and unfilled regions non-ancestral material. (A) The first event going backward in time is a recombination event. (B) The second event is another recombination event. (C) The third event is a coalescent event creating a new sequence, where the ancestral material is partitioned into two segments with non-ancestral material in between. This non-ancestral material is *trapped* between the two segments of ancestral material. (D and E) Coalescent events merge the ancestral material back onto a single genomic background. This figure was inspired by the original figure of Wiuf and Hein [30].
(TIF)

S2 Fig The inferred number of recombination events in ARGs sampled by ARGweaver with the maximum joint likelihood, maximum likelihood, and maximum iteration as compared to the true simulated numbers under different *μ*/*r* ratios. In the legend, *Max*_*iter*, *Max*_*Likeli*, *Max*_*Joint* represents maximum iteration, maximum likelihood, and maximum joint likelihood, respectively.
(TIF)

S3 Fig Scaled Kendall-Colijn (KC) distances between the true (simulated) local trees and the local tree inferred by ARGweaver in the reconstructed ARG under different ratios of mutation rate / recombination rate. The lambda value in the KC metric was set at either 0.0, 0.5, and 1.0, where higher lambda values preferentially weight branch length differences over topological differences. For each simulation, *N*_*e*_ is 100, sample size is 50, genome length is 1e04, and recombination rate *r* is 2.5e-06. Under each ratio, 100 simulations were run.
(TIF)

S4 Fig Average pairwise genetic diversity pi in sequences simulated under different *μ*/*r* ratios. (TIF)

S5 Fig Estimating migration rates M with different sample sizes. Estimating migration rates M between two subpopulations with (A) 20 samples, (B) 50 samples, and (C) 100 samples. Each black line is *x* = *y*. Dots and blue bars represent the median posterior estimates and the 95% confidence intervals for each simulation.
(TIF)

S6 FigEffective population size, recombination rates and migration rates estimated using SCAR from ARGs inferred by ARGweaver under different *μ*/*r* ratios.The dashed red lines are the simulated effective population size, recombination rate and migration rate in all simulations, respectively. For each simulation, genome length is 1e04, recombination rate *r* is 2.5e-06, and each population has two subpopulations, for each subpopulation *N*_*e*_ is 50, sample size is 25, migration rate is 0.015. Because ARGweaver assumes a single panmictic population, we scaled the effective population sizes $N_{e}^{{}^{\prime }}$ input into ARGweaver to be equivalent in terms of coalescent rates to that of a structured population with two demes using equation 4.22 in Rice [77]. Under each ratio, 100 simulations were run.(TIF)

S7 Fig ARG of the 51 lineage IB isolates (A), 48 lineage IC isolates (B) and the aflatoxin gene cluster of lineage IC (C) reconstructed by ARGweaver. The reconstructed ARG is visualized using a tanglegram to show how the topology of local trees varies across chromosome 3. Each local tree corresponds to one genome region separated from neighboring regions by an inferred recombination breakpoint. Note only the first 10 of 193 local trees in the ARG of lineage IB, and only the first 10 of 775 local trees in the ARG of lineage IC are shown. In the ARG of the aflatoxin gene cluster, there are 12 local trees.
(TIF)

S8 Fig RF distance between neighboring local trees, and average RF distance calculated from 20 one-random-SPR trees of lineages IB and IC, respectively. (TIF)

S9 Fig The relationship of genome location distance and RF distance of pairs of local trees, and the histogram of non-recombination segment length (neighboring breakpoints distance). When calculating the genome location distance, we set the middle location of each genome region as coordinates, and then the distance is the absolute value of coordinates difference between two trees.
(TIF)

S10 Fig The migration rates (per generation) likelihood profile of lineages IB and IC between subpopulations in four states using the SCAR model. (TIF)

S1 Table *A. flavus* isolates metadata. *A. flavus* isolates sampling locations, lineages, and other information for lineages IB and IC.
(XLSX)

S2 Table All runtime parameters of ARGweaver. The results of Watterson’s theta and population recombination rate calculated by LDhat at different missing threshold levels. The parameters used when running ARGweaver.
(XLSX)

S3 Table The posterior distribution of recombination and migration rates estimated from both the full ARG and the consensus tree. (XLSX)
