# Supplementary figures and images for: Recombination-aware phylogeographic inference using the structured coalescent with ancestral recombination
Source: PLoS Comput Biol. 2022 Aug 19;18(8):e1010422. doi: 10.1371/journal.pcbi.1010422 (PMC9447913; doi:10.1371/journal.pcbi.1010422)

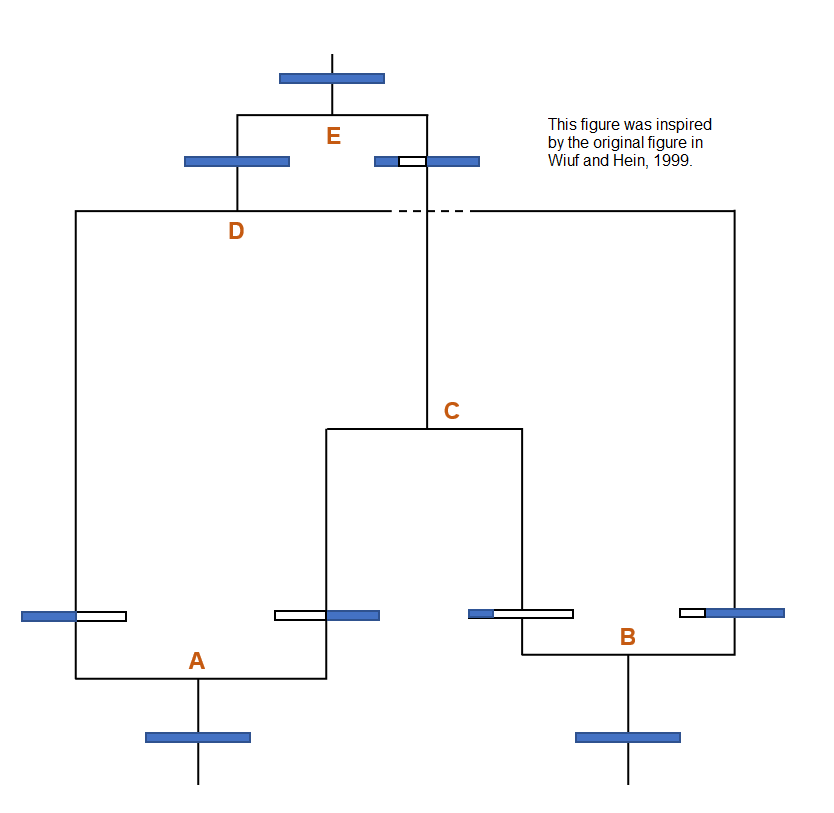

Supplement: S1 Fig — Time starts at present (bottom) and increases going backward in time (top). The genome of each lineage is represented by a rectangle with blue filled regions containing material ancestral to the sample and unfilled regions non-ancestral material. (A) The first event going backward in time is a recombination event. (B) The second event is another recombination event. (C) The third event is a coalescent event creating a new sequence, where the ancestral material is partitioned into two segments with non-ancestral material in between. This non-ancestral material is trapped between the two segments of ancestral material. (D and E) Coalescent events merge the ancestral material back onto a single genomic background. This figure was inspired by the original figure of Wiuf and Hein [30]. (TIF) [file pcbi.1010422.s001.tif]

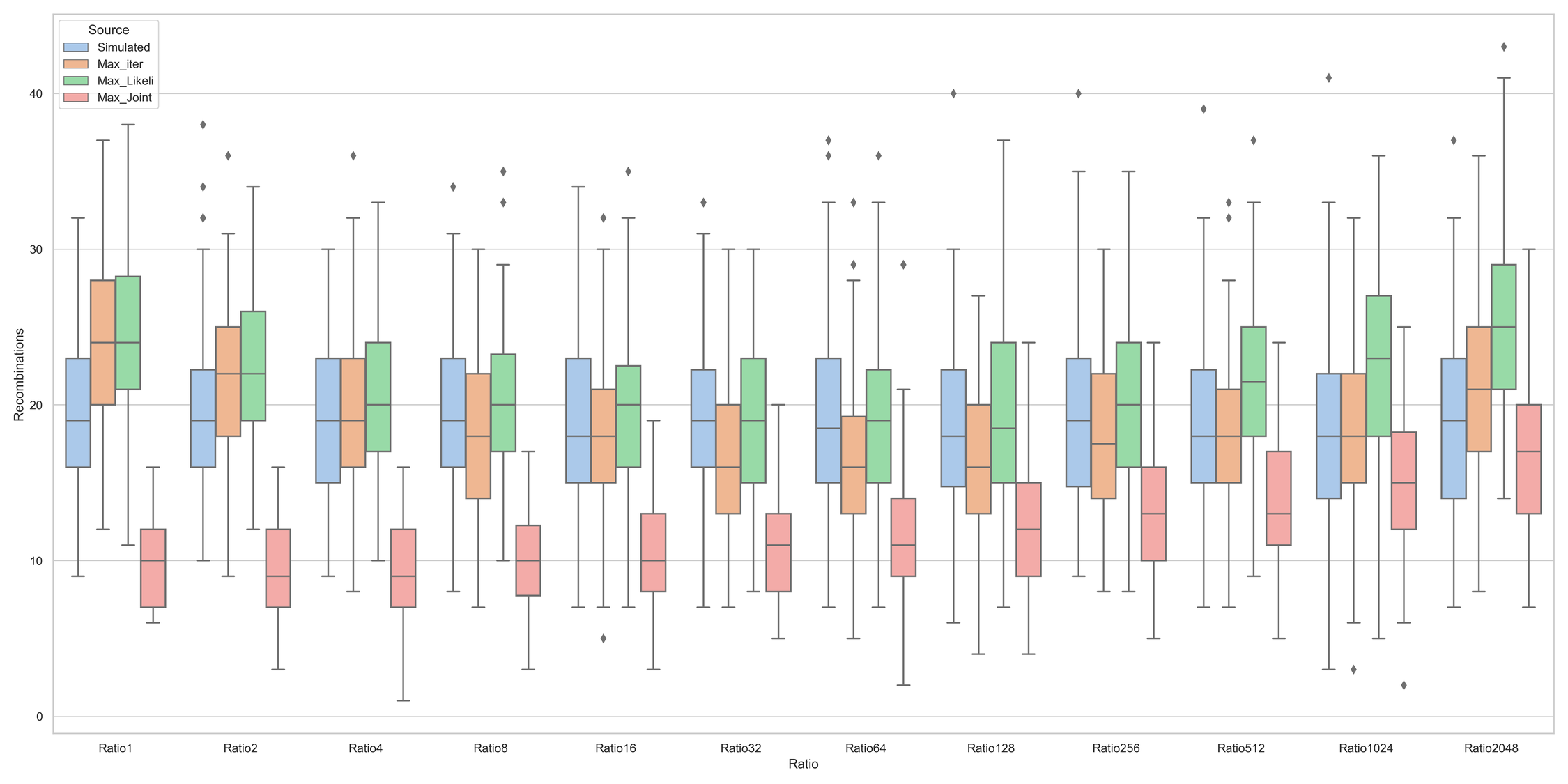

Supplement: S2 Fig — In the legend, Max_iter, Max_Likeli, Max_Joint represents maximum iteration, maximum likelihood, and maximum joint likelihood, respectively. (TIF) [file pcbi.1010422.s002.tif]

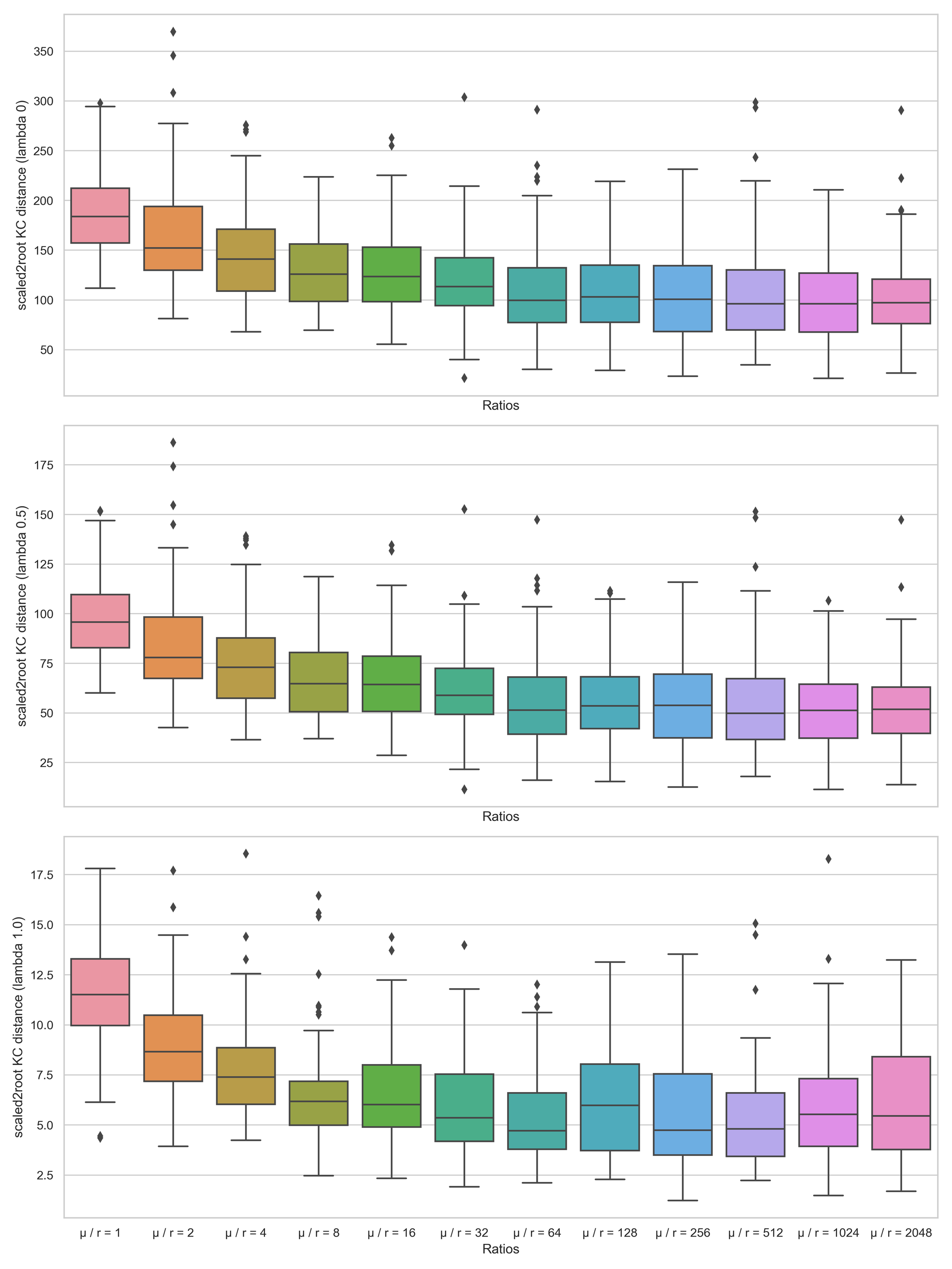

Supplement: S3 Fig — The lambda value in the KC metric was set at either 0.0, 0.5, and 1.0, where higher lambda values preferentially weight branch length differences over topological differences. For each simulation, Ne is 100, sample size is 50, genome length is 1e04, and recombination rate r is 2.5e-06. Under each ratio, 100 simulations were run. (TIF) [file pcbi.1010422.s003.tif]

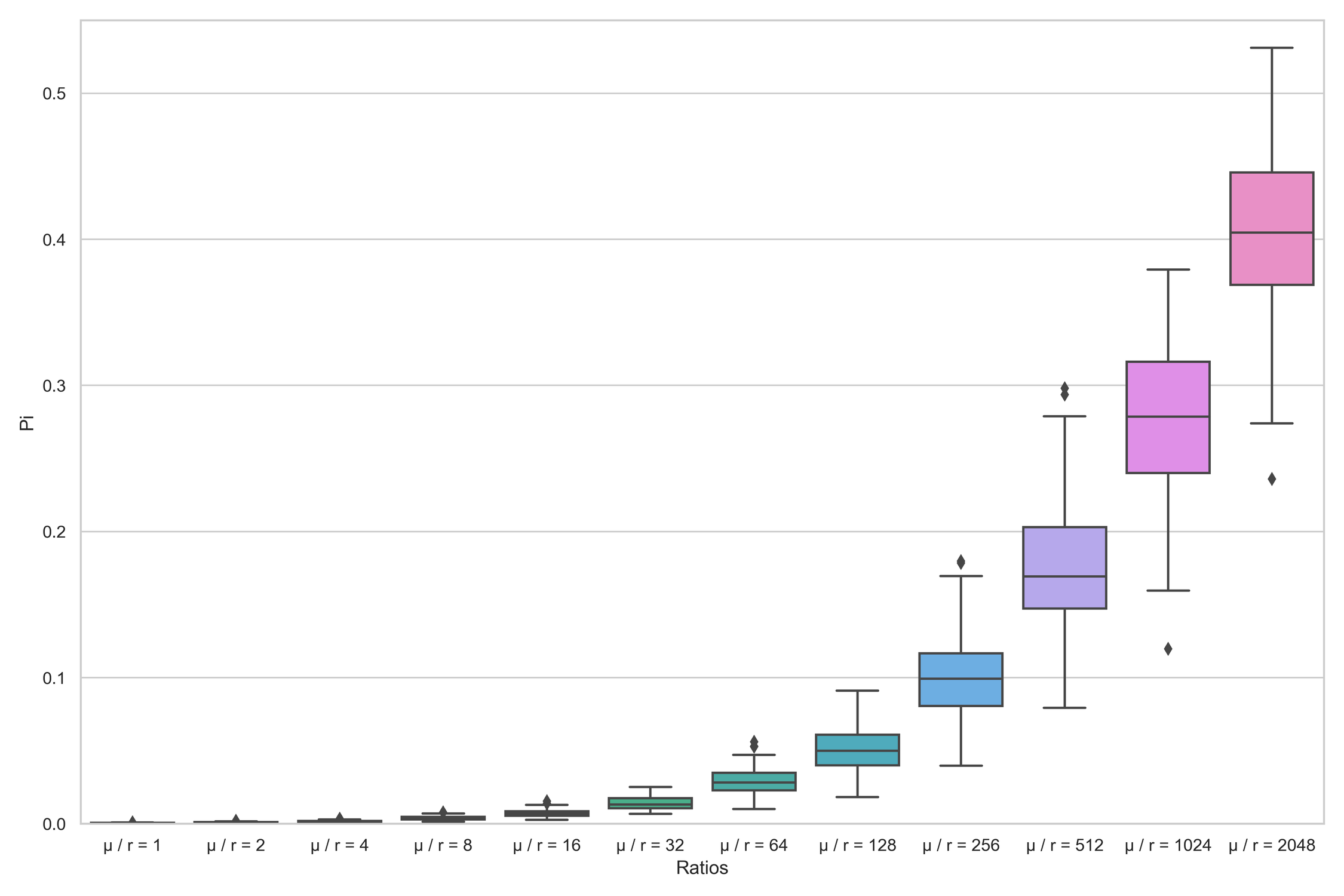

Supplement: S4 Fig — (TIF) [file pcbi.1010422.s004.tif]

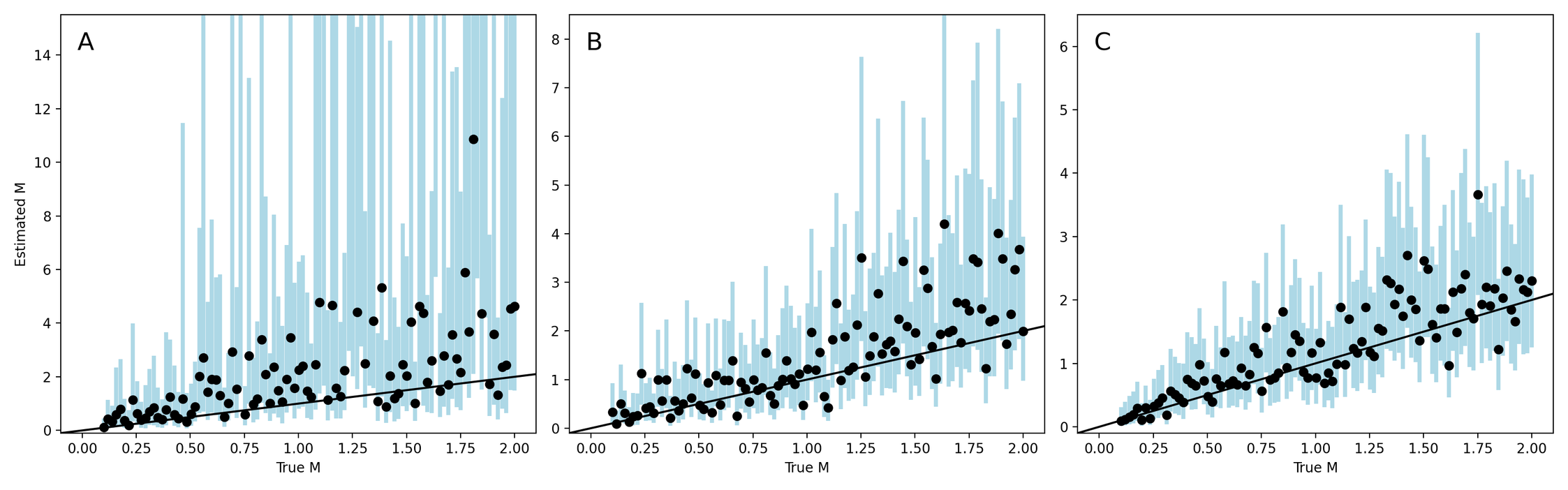

Supplement: S5 Fig — Estimating migration rates M between two subpopulations with (A) 20 samples, (B) 50 samples, and (C) 100 samples. Each black line is x = y. Dots and blue bars represent the median posterior estimates and the 95% confidence intervals for each simulation. (TIF) [file pcbi.1010422.s005.tif]

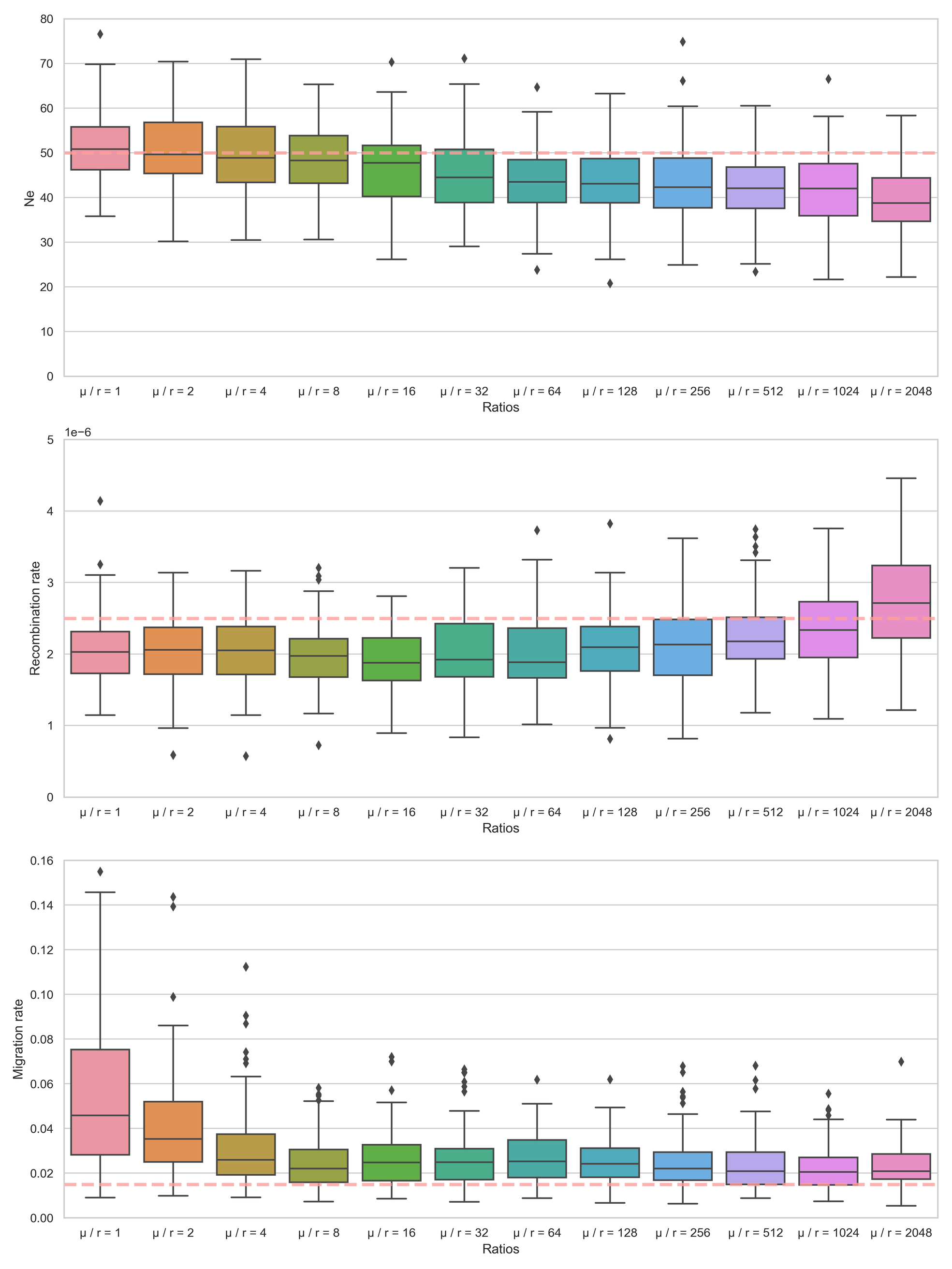

Supplement: S6 Fig — The dashed red lines are the simulated effective population size, recombination rate and migration rate in all simulations, respectively. For each simulation, genome length is 1e04, recombination rate r is 2.5e-06, and each population has two subpopulations, for each subpopulation Ne is 50, sample size is 25, migration rate is 0.015. Because ARGweaver assumes a single panmictic population, we scaled the effective population sizes Ne′ input into ARGweaver to be equivalent in terms of coalescent rates to that of a structured population with two demes using equation 4.22 in Rice [77]. Under each ratio, 100 simulations were run. (TIF) [file pcbi.1010422.s006.tif]

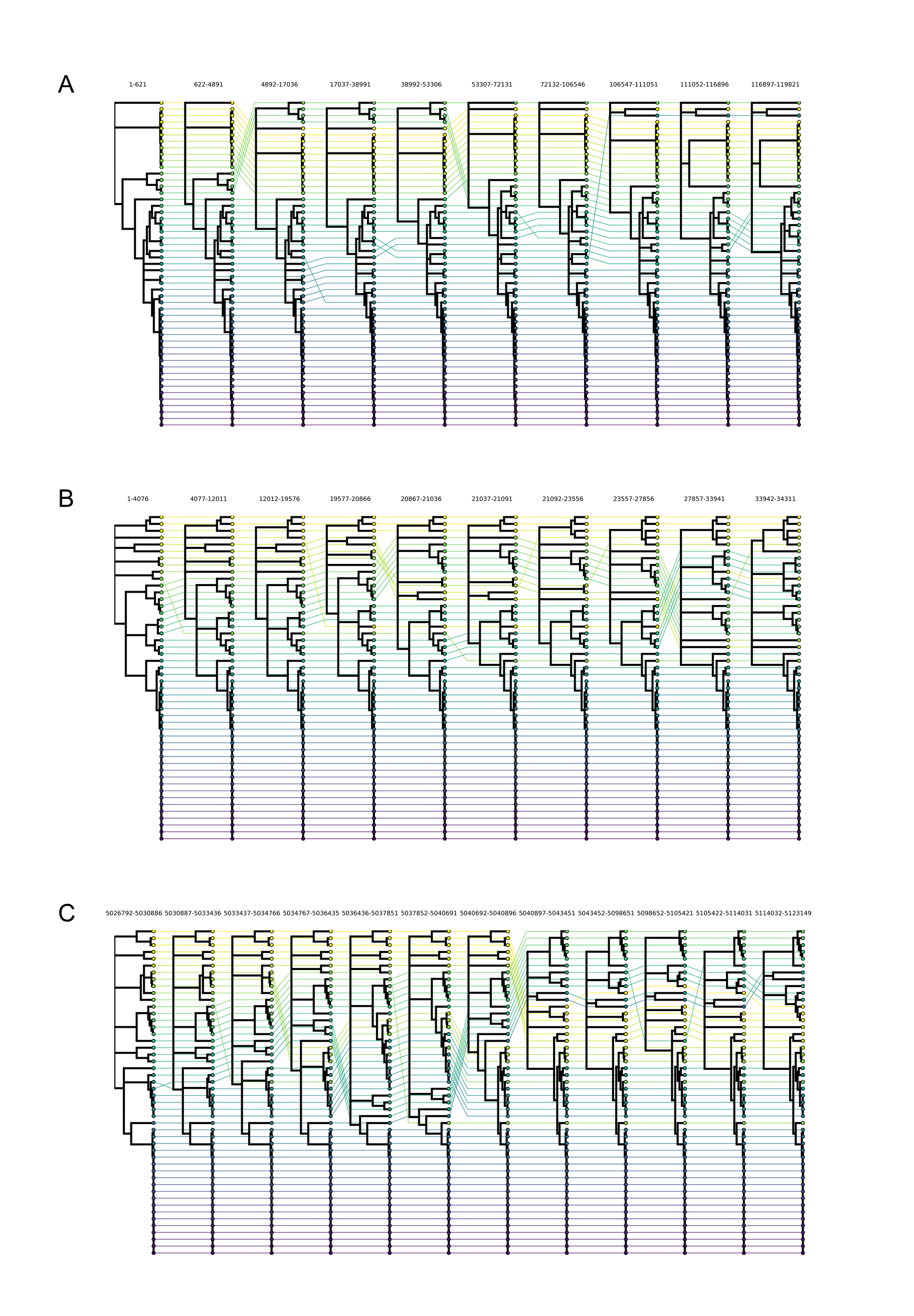

Supplement: S7 Fig — The reconstructed ARG is visualized using a tanglegram to show how the topology of local trees varies across chromosome 3. Each local tree corresponds to one genome region separated from neighboring regions by an inferred recombination breakpoint. Note only the first 10 of 193 local trees in the ARG of lineage IB, and only the first 10 of 775 local trees in the ARG of lineage IC are shown. In the ARG of the aflatoxin gene cluster, there are 12 local trees. (TIF) [file pcbi.1010422.s007.tif]

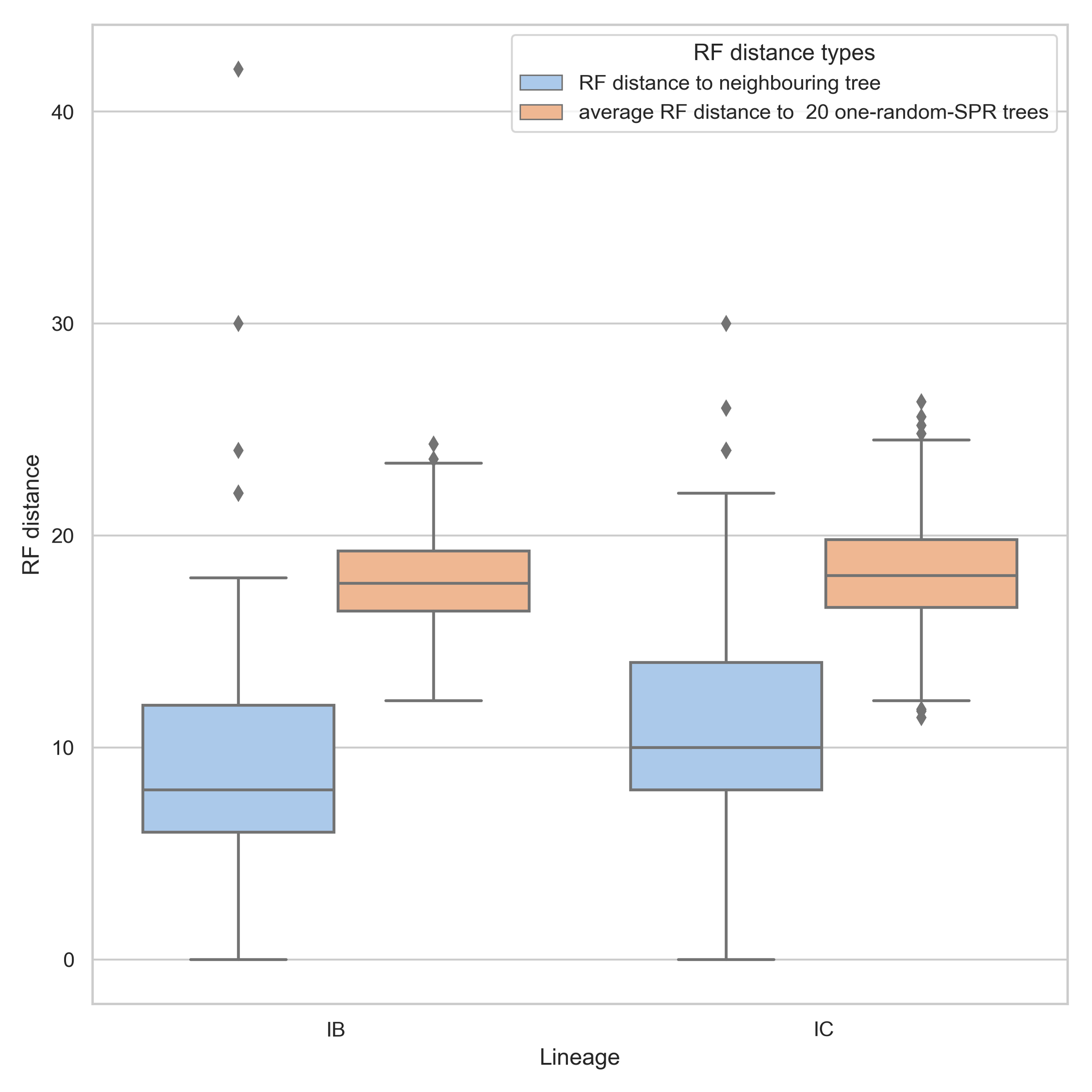

Supplement: S8 Fig — (TIF) [file pcbi.1010422.s008.tif]

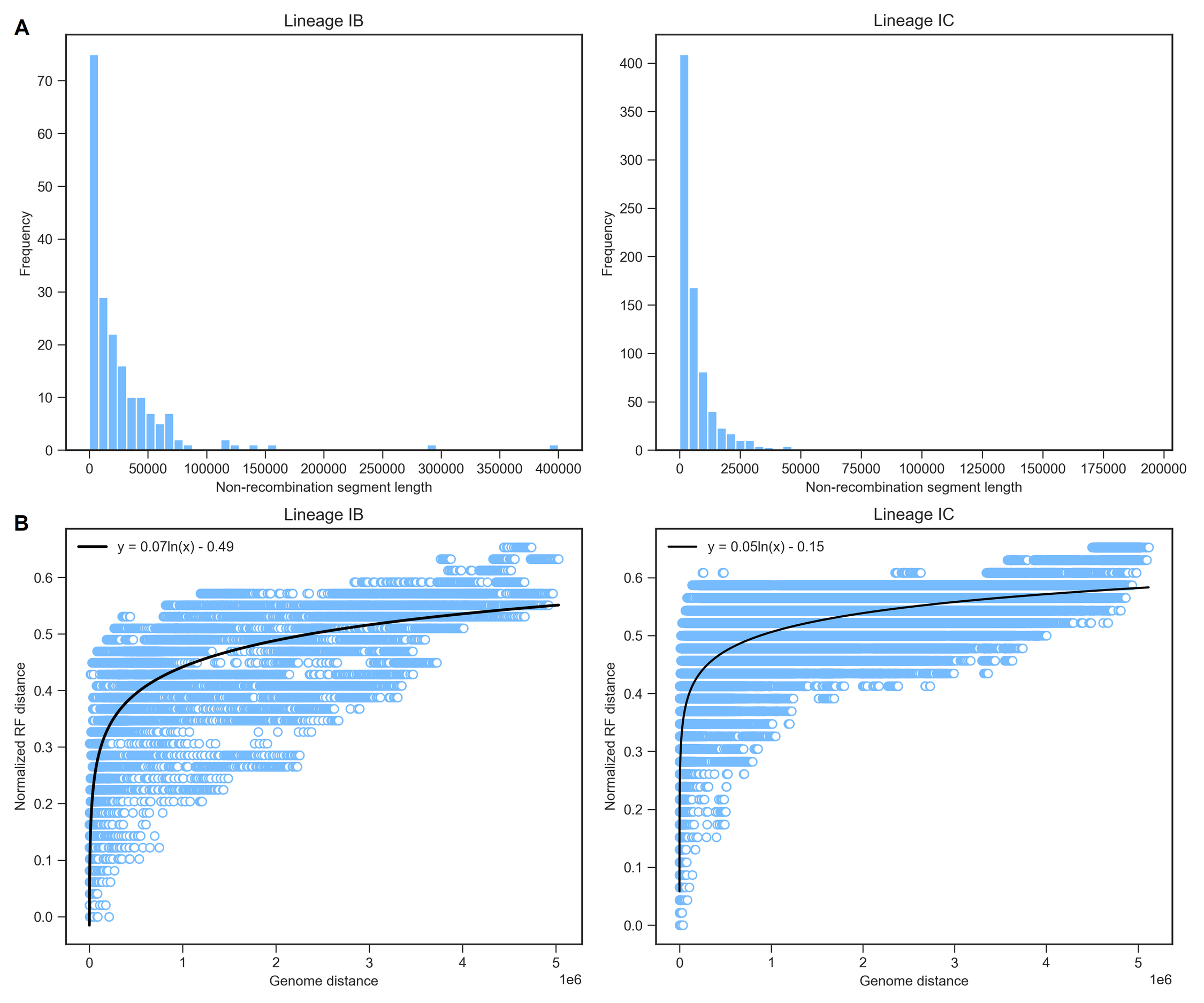

Supplement: S9 Fig — When calculating the genome location distance, we set the middle location of each genome region as coordinates, and then the distance is the absolute value of coordinates difference between two trees. (TIF) [file pcbi.1010422.s009.tif]

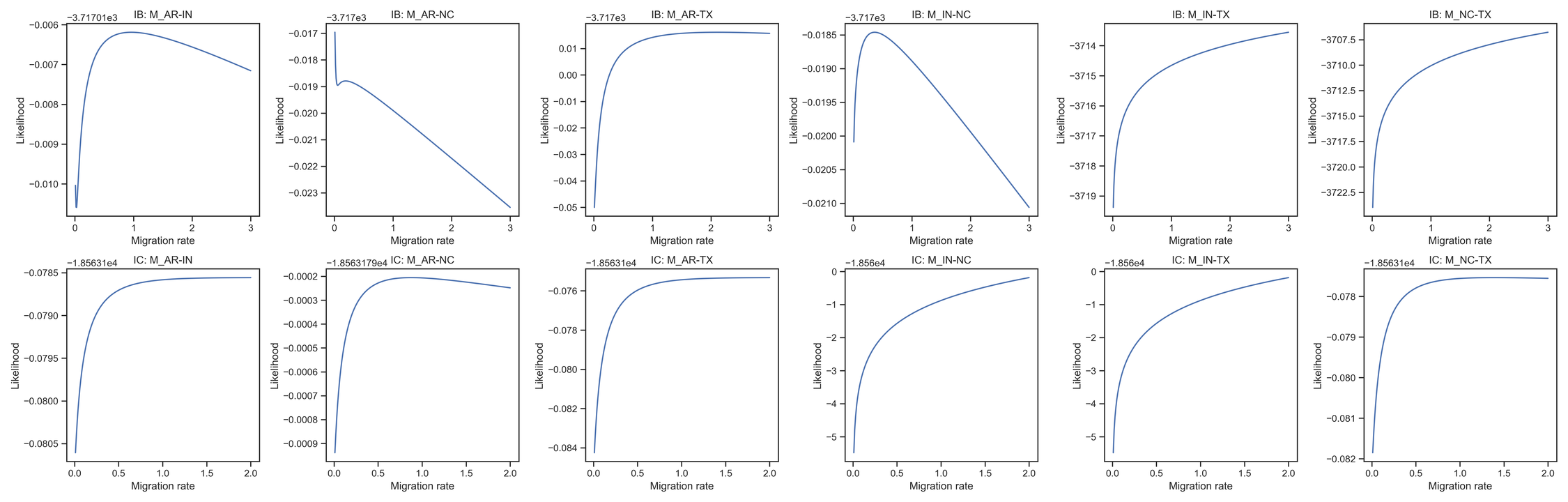

Supplement: S10 Fig — (TIF) [file pcbi.1010422.s010.tif]
